# Fifty years of maternal mortality research in sub-Saharan Africa: a bibliometric analysis of trends, gaps, and opportunities for health equity

**DOI:** 10.3389/fgwh.2026.1717410

**Published:** 2026-07-08

**Authors:** Modupe M. Fasina, Gaotswake P. Kovane, Ishaku L. Elisha, Angel Phuti

**Affiliations:** 1 Institute of International Health, Charité – Universitätsmedizin Berlin, corporate member of Freie Universität Berlin and Humboldt Universität zu Berlin, Berlin, Germany; 2 NuMIQ Research Focus Area, Faculty of Health Sciences, North-West University, Mahikeng, South Africa; 3 Drug Development Division, National Veterinary Research Institute, Vom, Plateau State, Nigeria; 4 Department for Gynaecology with Center for Oncology, Charité – Universitätsmedizin Berlin, corporate member of Freie Universität Berlin and Humboldt Universität zu Berlin, Berlin, Germany

**Keywords:** health equity, health policy translation, maternal mortality, research gaps, sub-Saharan Africa

## Abstract

**Background:**

Sub-Saharan Africa (SSA) continues to bear a disproportionate share of the global burden of maternal mortality (MM), despite decades of advocacy, research, and investment.

**Methods:**

A bibliometric analysis investigated the evolution, volume, scope, and impact of maternal mortality research in SSA from 1975 to 2024, and presents a 50-year overview of peer-reviewed research on maternal mortality across SSA, identifying trends, prolific contributors, research gaps, and thematic shifts over the studied years. Moreover, using the Scopus database and VOSviewer, 1,985 publications were analyzed for authorship, institutional output, funding patterns, and coauthorship networks.

**Results:**

The results revealed substantially increased publication output over time, particularly from Nigeria, South Africa, and Ethiopia, though the regions remain dependent on external funding and underrepresented in global high-impact journals (≤2.6% of global MM research outputs). There were some critical disparities in authorship, language, and thematic areas, including the translation of research findings into policy and practice. The most cited studies were grounded in subnational and underserved populations in SSA. The highest MM rates were found in Chad, Nigeria, and South Sudan (>1,000 deaths/100,000 live births), yet Chad and South Sudan have been studied in the fewest publications. Persistent causes of maternal mortality such as postpartum hemorrhage, hypertension in pregnancy, unsafe abortion, and infection have remained unchanged for decades, while health system and sociocultural barriers, including geographic inaccessibility, conflict and internal displacement, continue to worsen clinical outcomes, undermining progress.

**Conclusion:**

This study underscores the broader systemic inequities in SSA that are reflected through progress in MM research and its outputs. Moreover, it calls for intersectional approaches in health, equity-driven, and transdisciplinary research and highlights the need for regional ownership, particularly in underrepresented areas. Capacity building and strengthening local research ecosystems through investments in research infrastructure and cross-sectoral partnerships to bridge research–policy gaps are recommended. Context-driven solutions are crucial to make tangible progress. Such solutions are aligned with the attainment of the Sustainable Development Goals and national health security plans.

## Introduction

Maternal mortality (MM) is both a marker and determinant of national health. It remains a most urgent and persistent global health challenge and is also a key indicator of broader socioeconomic development (1). Defined as death during pregnancy or within 42 days postpartum, it remains unacceptably high in SSA, where the maternal mortality ratio (MMR) averages 542 deaths per 100,000 live births—over twice the global average (2). Despite international commitment and expanding research, sub-Saharan Africa (SSA) accounts for nearly 70% of global maternal deaths, with approximately 287,000–300,000 women dying annually (2–4).

Historically, multiple challenges in SSA have been shaped by colonial and historical legacies. For instance, the colonial era had a significant impact on the health systems of many African countries, perpetuating structural inequalities that continue to affect resource allocation, workforce distribution, and policy development (5–7). More recently, geopolitical instability, donor dependency, and global health emergencies have disrupted progress in building resilient health systems capable of reducing maternal deaths sustainably (8, 9).

Despite this context, and the additional fact that the WHO African Region continues to have the highest adolescent birth rate globally (approximately 120 per 1,000 adolescent women), maternal mortality research in sub-Saharan Africa appears inconsistent (10). Much of the existing literature focuses on clinical interventions, underemphasizing sociocultural, anthropological, and governance-related dimensions (11). Furthermore, it appears that research has not always been translated into sustainable policy action or community-level implementation (12). As global health shifts toward more equity-focused, integrated approaches—aligned with Sustainable Development Goal (SDG) 3.1, which aims to reduce the global MMR to below 70 deaths per 100,000 live births by 2030—there is a need to reassess how maternal mortality is studied, communicated, and addressed in policy and practice (13, 14). Furthermore, while research output on maternal mortality has surged in recent years, it is unclear whether it aligns with evolving risks, determinants, and policy needs. This study, therefore, aims to evaluate 50 years of maternal mortality research in sub-Saharan Africa, paying particular attention to evolving trends, shifting determinants, and the factors that enable or hinder the adoption of evidence-based solutions. It is expected that by synthesizing the trajectory of maternal mortality research and its real-world impact, the outcome should inform more holistic, intersectional, and accountable approaches to maternal health in the sub-Saharan African region to reduce avoidable deaths among women.

## Materials and methods

### Bibliometric analysis

**Figure 1:**
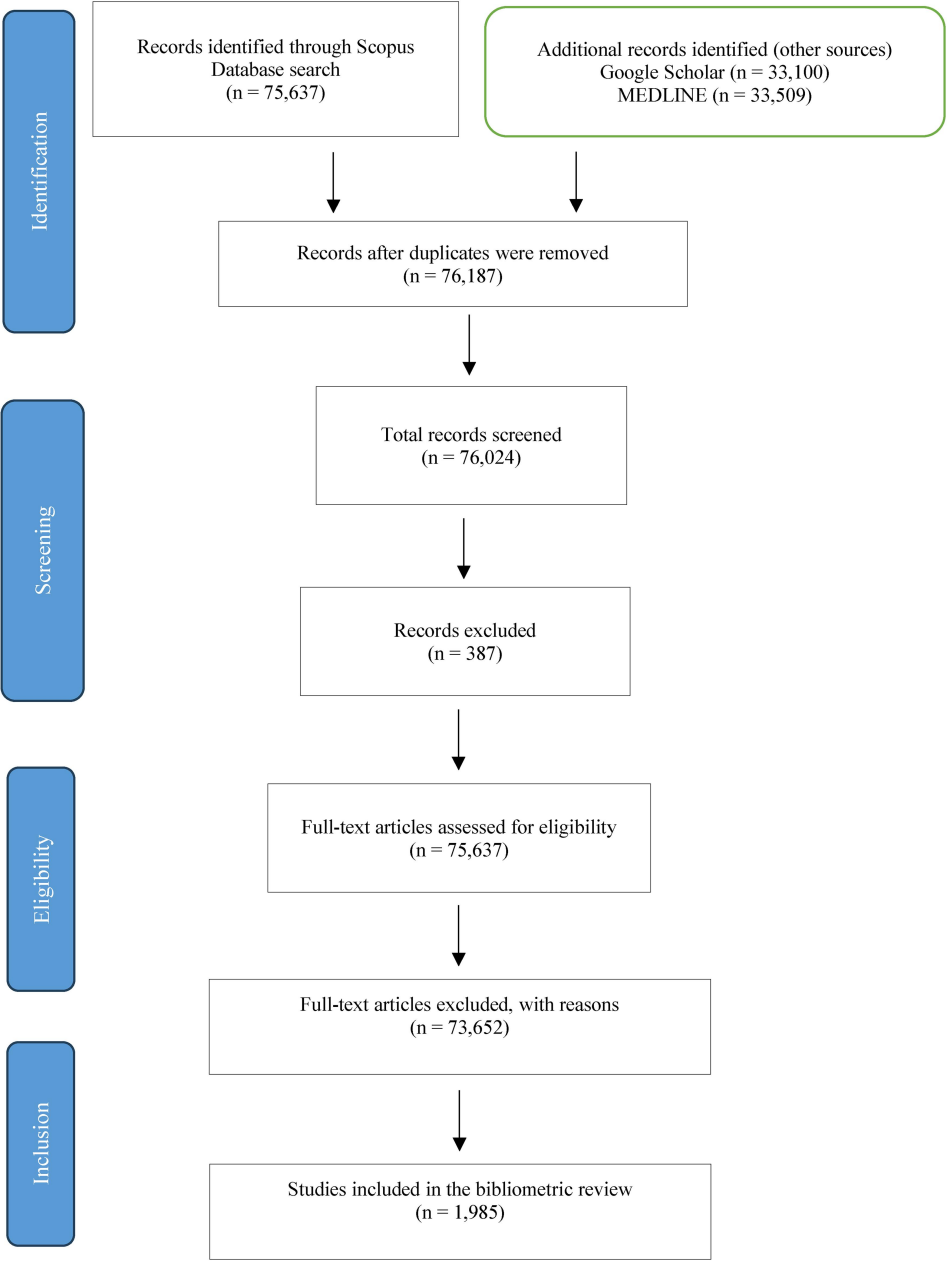
Modified PRISMA flow diagram of the study selection process (15).

A bibliometric review of peer-reviewed literature on maternal mortality in SSA from 1975 to 2024 was conducted using Scopus, and supported by retrievals from Google Scholar and MEDLINE [Figure 1 (15)].

### Justification for database selection

Scopus was selected as the primary database for this bibliometric analysis for the following reasons: (1) it is the largest abstract and citation database of peer-reviewed literature, with over 25,000 active titles from 5,000 publishers; (2) it offers superior coverage of African journals compared to Web of Science, with indexing of over 300 African journals; (3) its export functionality is fully compatible with VOSviewer for bibliometric analysis; (4) it provides comprehensive citation data for impact assessment; and (5) it includes substantial coverage of French and Portuguese language publications through its broader journal indexing compared to Web of Science. Google Scholar and MEDLINE were used as supplementary sources to identify any relevant studies not captured in Scopus, though their citation data were not used for bibliometric analysis due to known limitations (inconsistent citation counts and inclusion of non-peer-reviewed materials). Web of Science was not used as a primary database due to its more limited coverage of African journals and reduced compatibility with our analytical workflow, though we acknowledge this as a potential limitation (see Limitations section). The Cumulative Index to Nursing and Allied Health Literature (CINAHL) was not included as its focus on nursing and allied health literature was less central to our research questions on maternal mortality epidemiology and health systems.

### Search strategy and information sources

A bibliometric search was conducted using the Scopus database as the primary source, supplemented by Google Scholar and MEDLINE to ensure comprehensive coverage. The search was performed between 1 and 25 August 2024 and covered the period January 1975 to August 2024. The search strategy was developed using a broad topic-based approach to maximize the retrieval of relevant literature. Search terms were organized into the following three conceptual groups: (1) geographic scope (all 47 sub-Saharan African countries listed individually); (2) maternal mortality outcomes (e.g., “maternal mortality,” “maternal death,” and “pregnancy-related death”); (3) direct clinical causes (e.g., “postpartum hemorrhage,” “eclampsia,” and “unsafe abortion”); and (4) risk factors and determinants (e.g., “health system barriers,” “socio-cultural factors,” and “conflict”). Boolean operators (AND, OR) were used to combine terms. The full search string is provided in Supplementary File S1. This broad strategy was intentionally designed to capture the full spectrum of published research on maternal mortality and its determinants in sub-Saharan Africa, which is appropriate for a bibliometric analysis.

The broad inclusion of risk factor and determinant terms was intentional to capture the full spectrum of research on factors contributing to maternal mortality, not merely studies where maternal death was the primary outcome. This comprehensive approach explains the high initial hit count (76,187), which was subsequently refined through screening (see the modified PRISMA flow diagram in Figure 1).

The inclusion criteria were as follows: (1) focus on maternal mortality as a primary or secondary outcome; (2) study population located in one or more sub-Saharan African countries; (3) peer-reviewed original research, systematic reviews, or meta-analyses; (4) published between 1975 and 2024; (5) available in English, French, or Portuguese; and (6) sufficient bibliographic information for analysis (authors, affiliations, abstract, keywords).

The exclusion criteria were as follows: (1) editorials, commentaries, letters, conference abstracts, or opinion pieces without original data; (2) studies focused exclusively on maternal morbidity without mortality data; (3) studies conducted outside SSA; and (4) duplicate publications.

Gray literature (technical reports, policy briefs, and theses) was excluded from the bibliometric analysis to maintain consistency in citation metrics and quality standards. However, key policy documents from the WHO, UN agencies, and Ministries of Health were consulted for the Discussion section to contextualize findings. Non-indexed local journals not captured in Scopus were not included, which we acknowledge as a limitation (see Limitations section).

Given the bibliometric nature of this study, formal quality assessment or risk of bias appraisal of individual studies was not performed. Bibliometric analysis examines publication patterns and impact rather than the methodological quality of individual studies. However, for the synthesis of causes of maternal mortality (Table 1), we drew primarily from systematic reviews and multicountry studies with robust methodologies, as cited. Supplementary data on MMR trends were sourced from UN and WHO reports (2, 16).

**Table 1:** Risk factors, leading causes, complications, and contributions to maternal mortality.

Variable	Contribution to death	Mean (%)	Range (%)
Severe (sometimes ante-partum and often postnatal) obstetric hemorrhage (postpartum hemorrhage, PPH)	Direct	26.3	7.0–41.3
Postnatal infections and sepsis (PNIS)	Direct	15.2	4.4–28.3
Pregnancy-induced hypertension (PIH)	Direct	19.7	10.3–37.3
Perinatal complications during delivery (including dystocia and uterine rupture)	Direct	14.3	5.5–29.0
Unsafe abortion	Direct	17.5	6.8–36.0
Eclampsia/pre-eclampsia	Direct	17.3	6.0–23.6
Pre-existing medical condition/diseases	Direct	14.1	14.1
Psychotic conditions	Indirect	17.0	17.0
Cardiovascular diseases	Indirect	16.0	16.0
Hepatitis	Indirect	18.6	18.6
Anesthetic-related deaths	Indirect	14.6	14.6
Anemia in pregnancy	Indirect	12.9	10.4–14.6
Meningitis	Indirect	12.0	12.0
HIV/AIDS	Indirect	10.6	10.6
Acute renal failure	Indirect	8.0	8.0
Malaria	Indirect	3.6	3.6
Indirect/other disorders	Indirect	23.4	8.0–36.6

See Supplementary List 2 for details of the references in the table. Mean percentages calculated from studies cited in Supplementary References (1–16); ranges reflect variation across individual studies.

Citation counts were extracted directly from Scopus on 30 August 2024, using the “Citation Count” feature. For the most-cited studies analysis, we verified Scopus citation counts against Google Scholar to ensure consistency; where discrepancies exceeded 10%, we used the Scopus count as the primary metric given its standardized indexing. Citation data for NIH-funded studies were obtained through the NIH RePORTER tool and cross-referenced with Scopus for harmonization. All citation data reflect counts at the time of extraction; we acknowledge that citation metrics are dynamic and may have changed since data collection.

### Extraction of risk factors, causes of maternal mortality, and maternal mortality ratio in sub-Saharan Africa, 1990–2023

**Figure 2:**
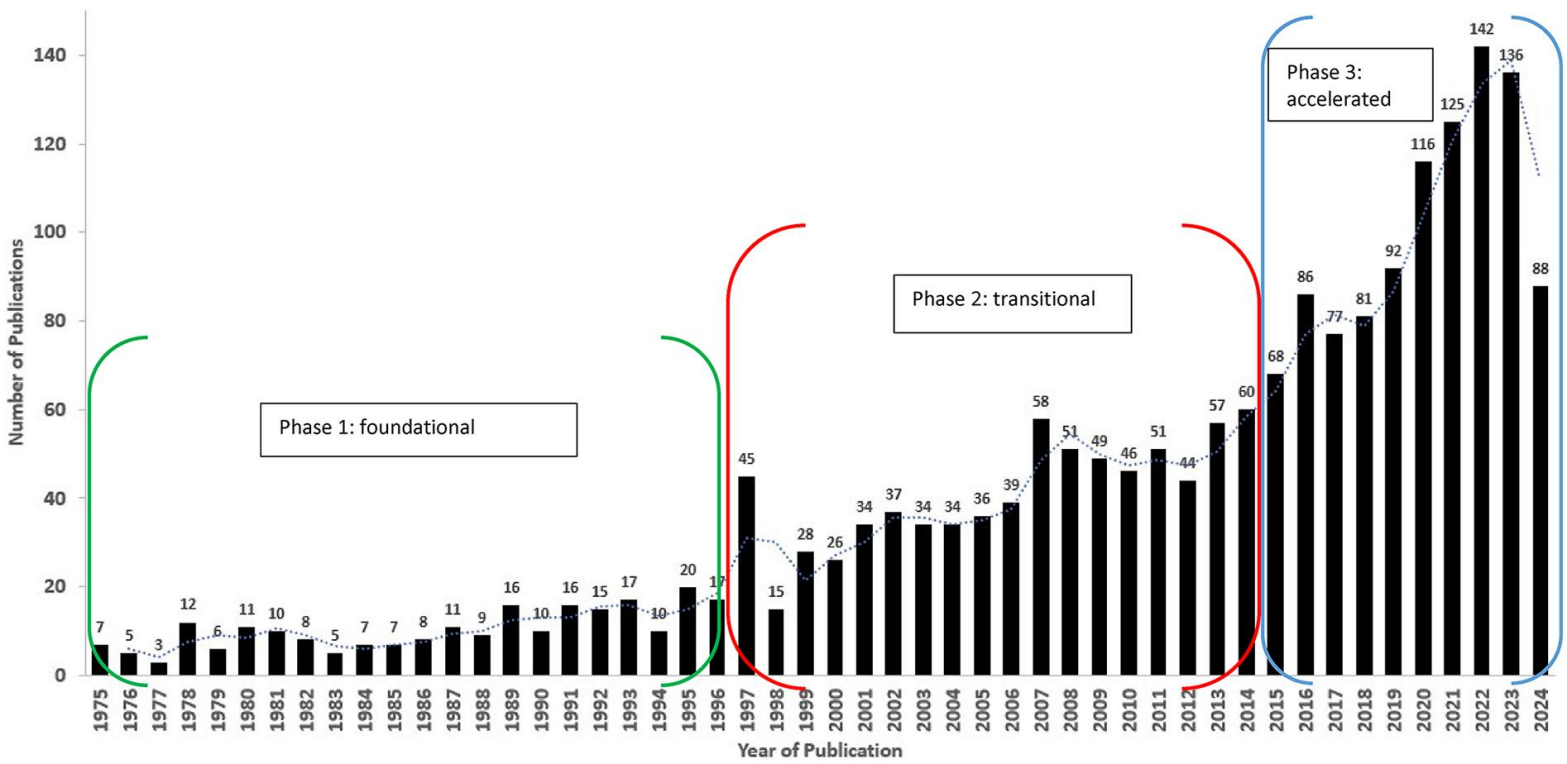
Temporal distribution of publications on maternal mortality in sub-Saharan Africa (1975–2024). The blue dashed line is the trendline of the 2-year moving average.

Based on the recent report of the United Nations Maternal Mortality Estimation Inter-Agency Group (MMEIG) and associated data (2, 16) and other resources (4), the estimated number of maternal deaths per 100,000 live births (MMR) for the 47 sub-Saharan African countries was retrieved using a 5-year series from 1990 up until 2023 (Figure 2; Supplementary Table S1). Peer-reviewed literature was also searched for risk factors, leading causes, complications, and contributions to maternal mortality (Table 2).

**Table 2:** Barriers and enablers of maternal mortality research and implementation in sub-Saharan Africa, 1975–2024.

Barrier and constraint	Enabler
Healthcare system weaknesses	Government commitment
Socioeconomic disparities	International partnerships
Policy inconsistencies	Community engagements
Hindering cultural norms	Technological innovations
Geographic inaccessibility	Health systems strengthening
Conflicts and internal displacements	Uptake of digital tools
Language barriers	Integration of community feedback loops
Underfunding and donor-reliance	

References for barriers and constraints (17–29); references for enablers (30–40).

### Calculation of cause-specific contributions to maternal mortality

The percentage contributions for direct clinical causes of maternal mortality, as presented in Table 2, were derived through a two-step process. First, we conducted a bibliometric review of studies reporting cause-specific maternal mortality in SSA populations (Supplementary List 2). Second, we calculated the mean percentage contribution of each cause across all included studies, weighting by study sample size where available. The range represents the minimum and maximum reported values across individual studies, reflecting geographic and temporal variation. The supplementary references listed at the bottom of Table 2 are the sources for these calculations; each contributed data to the pooled estimates.

### Data cleaning, curation, and analysis

All retained articles for bibliometric analysis (*n* = 1,985) were saved as *.csv (comma-separated values) and *.xlsx (Microsoft Excel 365 Spreadsheet) files and exported to VOSviewer for bibliometric analysis (41, 42). The bibliometric techniques employed by Elisha and Viljoen (43), and further refined in Elisha et al. (44), were used in the analysis with modifications and replacement of terms to suit research on MM (e.g., replacement of “rooibos tea” terms with “maternal mortality,” and retention of all bibliometric indicators). Bibliometric indicators (citation count, authorship, keywords, co-occurrences, thematic evolution, and institutional and funding affiliations) were analyzed using VOSviewer (42). Graphs and tables were constructed for journals, research, researchers, and countries based on citation, bibliographic coupling, cocitation, co-occurrence, and coauthorship relationships using the detailed analysis and outputs generated in VOSviewer (42).

**Figure 3:**
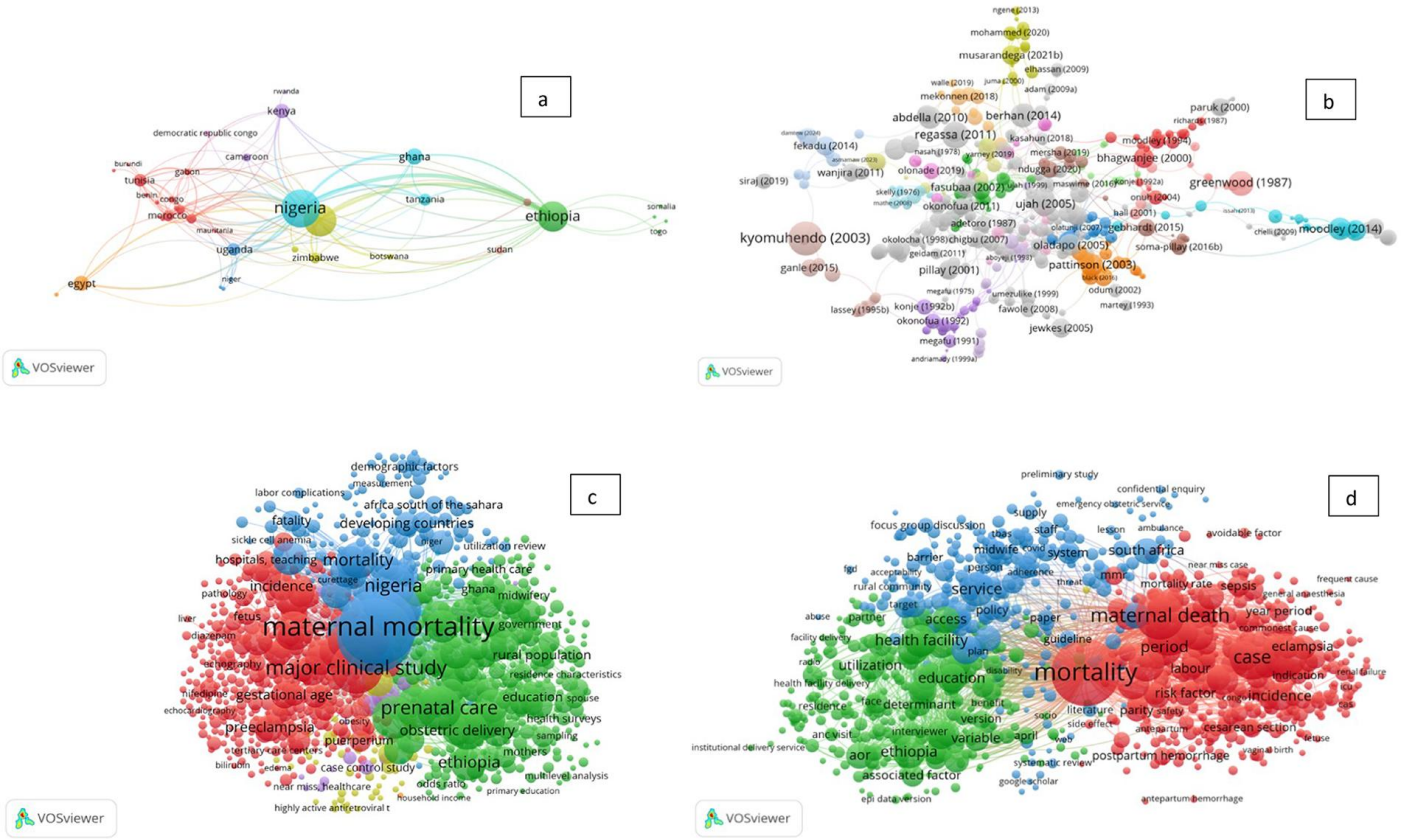
Temporal distribution of publications on maternal mortality in sub-Saharan Africa (1975–2024). **(a)** Peer-reviewed publications on maternal mortality from contributing sub-Saharan African countries; **(b)** lead author of peer-reviewed publications on maternal mortality in sub-Saharan Africa; **(c)** keywords used in peer-reviewed publications on maternal mortality research in sub-Saharan Africa; and **(d)** key terms used in peer-reviewed publications on maternal mortality research in sub-Saharan Africa.

In addition, due to the large size and density of the bibliometric dataset, which may reduce visualization clarity and interpretability in VOSviewer analyses (which is optimized for approximately 1,000 documents per analysis) (Centre for Science and Technology Studies, 2023), the 1,985 retrieved articles were divided into two temporal cohorts (1975–2014, *n* = 974; 2015–2024, *n* = 1,011) for separate network generation. The resulting maps were subsequently compared for thematic consistency and overlap and also compared with those of decade-wise analyses. The final network maps presented in Figures 3a–d and Supplementary Figures S1 and S2, therefore, represent an integrated and validated thematic structure derived from both bibliometric mapping and manual qualitative verification.

Google NotebookLM was used to assist in generating additional figures. All outputs were carefully reviewed, verified, and edited by the authors to ensure accuracy, originality, and compliance with journal standards.

## 3 Results

A total of 142,246 records were identified across Scopus (*n* = 75,637), Google Scholar (*n* = 33,100), and MEDLINE (*n* = 33,509). After removing duplicates (*n* = 66,059), 76,187 unique records were screened by title and abstract. Of these, 550 records were excluded as they did not address maternal mortality or were not focused on sub-Saharan Africa. The remaining 75,637 full-text articles were assessed for eligibility, of which 73,652 were excluded (reasons included not being focused on maternal mortality as the primary outcome, having been conducted outside SSA, or lacking original data). A total of 1,985 articles met the inclusion criteria and were included in the bibliometric analysis (Figure 1).

### Volume and trends in peer-reviewed publications by phase

A total of 1,985 articles were identified, showing exponential growth in the last decade. The trend in peer-reviewed publications was classified into the following three publication phases based on temporal evolution: foundational (1975–1996), transitional (1997–2014), and accelerated (2015–2024) (Figure 2). The modal year was 2022, coinciding with heightened global health investment in maternal mortality research and interventions. More specifically, this acceleration represents global health priorities, such as the Millennium Development Goals (MDGs) and Sustainable Development Goals, which galvanized attention and funding for critical issues, including maternal health. Notably, publication surges aligned with intensified local and international funding, policy reforms, and the expansion of open-access journals.

### Geographic and linguistic patterns (country contributions and language distribution)

Nigeria (*n* = 534), South Africa (*n* = 385), and Ethiopia (*n* = 343) emerged as the leading contributors to maternal mortality research by volume, accounting for over 50% of all publications (Figures 3a,b). South African institutions, such as the University of Witwatersrand and University of KwaZulu-Natal, and institutions in Nigeria and Ethiopia, such as the University of Nigeria and University of Gondar, were the most prolific institutions (Supplementary Table S3). English was the dominant language (over 90%), though substantial French-language output was noted from Francophone countries (*n* = 184). The underrepresentation of non-English research signals a critical equity gap in global knowledge dissemination (17, 18).

### Thematic evolution of research (clinical causes, access/utilization, health systems, and sociocultural barriers)

Keyword analysis using VOSviewer revealed the following three primary clusters: clinical/pathological research (Red bubbles), sociodemographic analyses (Blue bubbles), and health systems/policy interventions (Green bubbles) (Figure 3c). Common themes included clinical causes (e.g., postpartum hemorrhage, sepsis), access to care, and health system barriers. Keyword mapping revealed a shift from clinical focus (1975–1994) to systems and policy research (2015–2024) (Figure 3d, Supplementary Figures S1a–e). The patterns and shift in keywords and thematic evolution in maternal research reflect the maturation of the field toward more interdisciplinary and systems-based inquiry.

### Determinants, barriers, and enablers (integrated synthesis)

Over the 50-year period, the enablers of maternal mortality research included the following, among others: commitment from the national and subnational authorities, international partnerships, whole-of-society community engagement, adoption of technological innovation, and health system strengthening. The uptake of digital tools and integration of community feedback emerged as particularly promising trends (Table 1). Similarly, the barriers and constraints included unattended health system weaknesses; socioeconomic disparities; policy inconsistencies; cultural norms that are at variance with best practices in the health system; geographically difficult areas to access, which are prevalent in a number of sub-Saharan African countries; conflicts and internal displacement, as exemplified in Sudan, Democratic Republic of the Congo, and parts of Ethiopia, Nigeria, and South Sudan; language barriers; underfunding; and over-reliance on donor funding (Table 1).

### Temporal trends in maternal mortality research (1975–2024)

The analysis of publications across the three identified phases revealed distinct thematic shifts. During the foundational phase (1975–1996), research focused predominantly on clinical causes and hospital-based case series, with limited attention paid to community-level factors. The transitional phase (1997–2014) coincided with the Millennium Development Goal era, marked by increased focus on health system factors, skilled birth attendance, and emergency obstetric care. The accelerated phase (2015–2024) demonstrates greater methodological diversity, including implementation science, health systems strengthening research, and growing attention paid to socio-cultural determinants and health equity (Figure 2).

Regarding causes of maternal death, while the same clinical categories persist, their proportional contributions have shown subtle shifts. Bibliometric review evidence indicates that hemorrhage remains the leading cause (26.3%), though deaths from unsafe abortion have declined in several countries with policy reforms, while indirect causes (HIV, malaria, and non-communicable diseases) have gained recognition in the literature since 2010 (Table 2).

### Estimates and epidemiology (maternal mortality ratio trends and causes of death)

**Figure 4:**
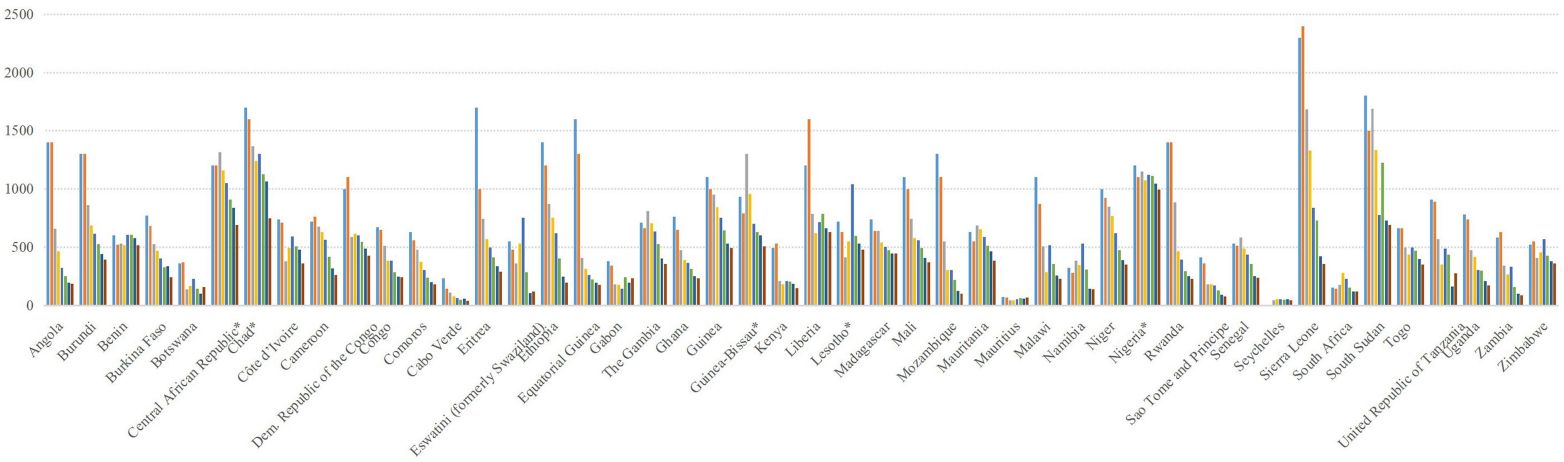
Five-year trend of point estimate decline in maternal mortality ratio in sub-Saharan Africa, 1990–2023. Data for 1990 and 1995 were retrieved from Estimates from Trends in Maternal Mortality: 1990 to 2013.^21^ Data for 2000–2023 were retrieved from the Internationally Comparable MMR Estimates report by the Maternal Mortality Inter-Agency Group.^2,24^ Data for earlier years (1975–1985) were unavailable. *Where MMR is rounded to the nearest 1 in the 2025 version of the MMEIG document, absolute values in the 2023 version were used. Countries with such values are asterisked.

Despite reductions over time, SSA remains the region with the highest MMR. In particular, our study found 17 countries with MMRs exceeding 1,000 deaths per 100,000 live births in 1990, and by 2020, Chad, Nigeria, and South Sudan still reported ratios above this threshold. By 2023, only Nigeria remained at 993 deaths per 100,000 live births (Figure 4). The direct clinical causes remained consistent across the 50-year period: (a) severe (sometimes ante-partum and often postnatal) obstetric hemorrhage (postpartum hemorrhage, PPH) (26.3%; range: 7.0–41.3%), (b) pregnancy-induced hypertension (PIH) (19.7%; range: 10.3–37.3%), (c) unsafe abortion (17.5%; range: 6.8–36.0%), (d) eclampsia/pre-eclampsia (17.3%; range: 6.0–23.6%), (e) peri-natal complications during delivery (including dystocia and uterine rupture) (14.3%; range: 5.5–29.0%), (f) postnatal infections and sepsis (PNIS) (15.2%; range: 4.4–28.3%), and (e) pre-existing medical condition/diseases (14.1%) (Table 2).

### Influential publications, journals, and authors

The most-cited works were subnational and context-specific studies (Supplementary Table S4), with Kyomuhendo leading in citations, followed by Ujah et al. and Regassa (45–47). The majority of these appeared in international journals such as *Reproductive Health Matters* and *BMC Pregnancy and Childbirth*, indicating that rural- and community-based studies gain significant attention when published in widely indexed platforms (Figure 4). Yet, leading African journals such as the *South African Medical Journal* and *Pan African Medical Journal* also played critical roles (Supplementary Table S5).

### Funding patterns and analysis

Between 1975 and 2024, a number of maternal mortality research studies have been funded. The lead funders, based on our analysis, included the Carnegie Corporation of New York (*n* = 19), the South African Medical Research Council (SAMRC) (*n* = 14), the Bill and Melinda Gates Foundation (BMGF) (*n* = 13), University of Gondar (*n* = 13), and the World Health Organization (*n* = 13) (Supplementary Table S5). Other funders are listed in the Supplementary Table S6.

### Interpretation of funding patterns

The funding landscape reveals several important patterns. First, sustained influence is evident from the Carnegie Corporation of New York (*n* = 19 funded publications across four decades), the South African Medical Research Council (*n* = 14, with increasing contributions since 2000), and the Bill and Melinda Gates Foundation (*n* = 13, primarily since 2010). These funders have supported longitudinal research programs and capacity building.

Second, thematic funding gaps are apparent. While obstetric hemorrhage and hypertension research receive consistent funding, sociocultural determinants, mental health aspects of maternal mortality, and health system governance receive disproportionately less support relative to their documented importance. Geographically, conflict-affected countries (Chad, South Sudan, and the Central African Republic) and fragile states remain severely underfunded despite having the highest MMRs.

Third, the participation of local African funding bodies has increased notably since 2015. South African institutions (SAMRC, University of the Witwatersrand, University of KwaZulu-Natal, University of Pretoria, University of Venda, and North-West University) and Ethiopian universities (University of Gondar, Jimma University, Addis Ababa University, Haramaya University, Hawassa University, and Mekelle University) now feature prominently. However, the funding landscape remains predominantly donor-dependent, with 78% of acknowledged funding originating from international sources (Global North institutions, bilateral aid, or philanthropic foundations based outside Africa). This dependency raises questions about research agenda ownership and sustainability, particularly given recent geopolitical shifts affecting global health funding.

## Discussion

While the volume of research has grown, the pace has fallen behind that of other regions, and its impact on policy and practice remains limited, with persistent gaps (48). It is thus necessary to prioritize region-specific research on maternal mortality to address gaps in data quality, availability, and relevance (49). Sub-Saharan Africa needs to strengthen its local research capacity, a critical factor in producing high-quality, context-specific studies that can inform policy and practice on the continent (50, 51). The significant growth in the literature on maternal mortality in SSA since the late 1990s is not unsurprising because it indicated the period with increased global and regional focus on maternal health issues (3, 52). In this period (late 1990s to early 2000s), major global health initiatives were launched, such as the United Nations MDGs in 2000, which included a specific target (MDG 5) to reduce maternal mortality by 75% by 2015 (53). These initiatives spurred maternal health research and associated funding for high-burden regions such as SSA (54) but may have promoted non-contextual, non-region-specific research, as mentioned above.

The same clinical drivers continue to cause deaths, indicating that research alone has not led to transformative change. This evidence supports previous findings by Say et al. (55) and Musarandega et al. (56) and highlights persistent clinical risks despite decades of intervention.

It should be understood that maternal mortality in SSA is not solely a consequence of biomedical complications, but also a reflection of broader inequities in health systems, governance, and socioeconomic development (55, 56). SSA countries must be intentional in reducing determinants of inequities, such as unequal access to quality health services, underserved poor areas, humanitarian crises, conflict, political instability, health workforce shortages, and underinvestment in rural healthcare infrastructure (30, 57). There should be cross-country learning of good practices in the achievement of the SDG 3 MMR target in SSA (especially from Cabo Verde, Mauritius, and Seychelles, which have surpassed the goals, and those that are progressing, such as Mozambique, Sao Tome and Principe, and Zambia) to significantly mitigate MM in this region (2, 58). There is also a need for a stronger focus on implementation and translational research, particularly in underrepresented countries such as Chad, South Sudan, and Sierra Leone. Health authorities should take advantage of the identified enabling factors in this work to push toward the aim of SDG 3.1, i.e., reducing the global maternal mortality ratio to less than 70 deaths per 100,000 live births by 2030. Sayinzoga et al. (33) and Maponga et al. (35) have identified similar drivers of improved health systems in resource-constrained countries in sub-Saharan Africa.

### Evolution of funding patterns and research priorities

The temporal analysis of funding acknowledgments revealed important shifts. In the 1990s, funding was predominantly from philanthropic foundations (the Carnegie Corporation and Rockefeller Foundation). The 2000s saw increased bilateral aid [the Department for International Development (DFID) and the United States Agency for International Development (USAID)] and multilateral agency funding [WHO and the United Nations Population Fund (UNFPA)]. From 2015 onward, we observe greater diversification, with African institutional funding (SAMRC and University of Gondar) becoming more prominent, though international donors still dominate. This evolution mirrors a global health priority shift from safe motherhood initiatives to MDGs and now SDG-focused investments.

### Caveats in funding interpretation

We acknowledge that funding acknowledgment data in publications provide an incomplete picture of the overall research funding landscape. They only capture projects that resulted in peer-reviewed publications, potentially underrepresenting implementation research, operational research, and capacity-building investments that may not lead to publications. Additionally, acknowledgment practices vary across journals and funders, and multiple funding sources for single studies complicate attribution. Therefore, our funding analysis should be interpreted as indicative of publication-supported research investments rather than a comprehensive mapping of all maternal health funding in SSA.

Although maternal mortality is primarily a clinical term, its causes and consequences are rooted in complex socioeconomic and political realities, as outlined above (59). Distant determinants, such as education, income, legal autonomy, and community wealth, influence more immediate factors, such as nutrition, reproductive health status, and access to care (60, 61). These determinants, in combination, create cumulative layers of risk that make pregnancy especially perilous for many women across sub-Saharan Africa. Additionally, the consequences of maternal death, ranging from economic hardship and child malnutrition to psychosocial trauma and community-level stigma, extend far beyond the individual who is directly involved (62, 63).

### Progress and persistent challenges in reducing maternal mortality

It is important to acknowledge the substantial progress achieved in reducing maternal mortality across SSA over the study period. Between 2000 and 2023, the global maternal mortality ratio declined by approximately 40%, with several SSA countries demonstrating remarkable progress (16). Sierra Leone, for example, reduced its MMR from 1,603 deaths per 100,000 live births in 2000 to 354 in 2023—a 78% reduction. Similar progress was evident in Ethiopia (from 953 to 195), Rwanda (from 1,007 to 229), and Zambia (from 630 to 85) (16, Supplementary Table S1). These reductions reflect the successful implementation of evidence-based interventions that increased skilled birth attendance, expanded access to emergency obstetric care, improved management of hemorrhage and hypertension, and, in some countries, led to safer abortion services.

However, despite this progress, the same clinical categories continue to contribute substantially to maternal deaths, indicating that research alone has not led to transformative change in all settings. As Say et al. (55) and Musarandega et al. (56) documented, obstetric hemorrhage remains the leading cause, though deaths from unsafe abortion have declined at a slower pace in countries with restrictive abortion laws. The persistence of these causes reflects implementation gaps—the “know-do gap”—rather than failure of biomedical knowledge. Effective interventions exist; the challenge lies in ensuring their equitable availability, accessibility, and quality across all populations, particularly in rural, conflict-affected, and underserved areas.

This work also confirmed that the local capacity remains low, and regional journals are underutilized, but at the same time, champions and mentors were identified. There is a need for the current generation of lead researchers, as identified in this work, to train the next generation of researchers. SSA requires investment in training and mentorship programs in order to build the capacity of early-career researchers in this region, including the provision of opportunities for advanced education, research funding, and professional development. There must be facilitation and promotion of South-South cooperation to share knowledge, resources, and best practices.

The persistent reliance on international funding and weak intra-African collaboration constrain sustainability. Equity-focused research is essential and should target conflict zones, rural areas, and marginalized populations. Strengthening research infrastructure and promoting open-access African journals are critical steps forward to the next phase of MM research in SSA. For instance, recent political decisions by the President of the United States of America, President Donald Trump, have significantly impacted health outcomes, including maternal health outcomes in sub-Saharan Africa; hence, the continent must strengthen its resilience to external shocks (64, 65). Finally, there is a need to invest more in translational research and increase subnational and non-English outputs. Moreover, interdisciplinary approaches, involving economists, anthropologists, and public health experts, are scarce but necessary.

Ultimately, reducing maternal mortality in SSA requires a whole-of-society approach. Policies must prioritize equity, gender inclusion, and context-responsive care. Health professionals, researchers, and policymakers must collaborate across disciplines to align data, evidence, and resources with community needs. By leveraging the knowledge built over five decades, SSA has the opportunity to develop resilient, inclusive, and accountable systems that protect maternal health and advance the global goal of ending preventable maternal deaths.

Understandably, influential authors such as Kyomuhendo, Ujah et al., and Regassa published their work in high-impact international journals and regional African journals, which were selected for their broad reach and indexing, in order to amplify the reach and visibility of context-specific studies from SSA. Specifically, Kyomuhendo's work on rural maternity services in Uganda appeared in Reproductive Health Matters, a journal with a strong focus on equity and policy, aligning with the socio-cultural themes of her research (45). Ujah et al.'s study on maternal mortality in Nigeria was published in the *African Journal of Reproductive Health*, reflecting its regional relevance and clinical focus (46), and Regassa's population-based study in Ethiopia was featured in *African Health Sciences*, emphasizing local data utility (47). The choice of journals reflects a dual strategy: targeting global platforms for wider citation impact while leveraging regional journals to address local policymakers and practitioners. However, the dominance of English-language publications (90%) underscores a gap in disseminating research from Francophone Africa, potentially limiting its policy uptake in those regions.

In addition, this bibliometric analysis underscores how maternal mortality in SSA is intertwined with broader geopolitical challenges. First, regarding equity and power imbalances, the reliance on external funding (e.g., from Global North institutions) shapes research priorities, which are often misaligned with local needs. This article calls for “regional ownership” to counter this dependency. Second, conflicts, instability, and weak governance in countries such as South Sudan and Chad, countries with the highest MMRs, highlighted how geopolitical instability exacerbates health disparities. Third, this article noted that surges in research output coincided with global initiatives. Finally, success stories from countries such as Cabo Verde and Ethiopia can assist in reducing reliance on Western-led frameworks and funding. Furthermore, this work reveals critical insights about funding. Underrepresented countries (e.g., Chad and Sierra Leone) lack resources to produce or disseminate research, meaning a few countries dominate the discourse in this field. A large proportion of the current funding prioritizes “clinical interventions” over sociocultural or governance research, thus limiting holistic solutions. Similarly, we have emphasized that donor-driven projects often fail to address local context, perpetuating inequities (66–68).

### Limitations

This study has several limitations that should be considered when interpreting findings. First, database bias: The primary use of Scopus, while justified by its comprehensive coverage and analytical compatibility with VOSviewer, may underrepresent research published in non-indexed regional journals, non-English language publications, and gray literature. It should be acknowledged that the search was conducted in English only, but French/Portuguese publications were included *post hoc*, if identified. This likely resulted in underrepresentation of Francophone and Lusophone SSA countries, where research may be published in French or Portuguese in local journals not captured by Scopus. Our finding that >90% of publications are in English confirms this bias.

Second, publication bias: Studies with statistically significant findings or those from well-resourced institutions are more likely to be published and cited, potentially skewing the evidence base toward certain settings or findings. The underrepresentation of conflict-affected countries (Chad, South Sudan, and the Central African Republic) in the publication record, despite their high MMRs, exemplifies this bias.

Third, data inconsistency across time and sources: Maternal mortality ratio estimates vary between WHO/MMEIG, Global Burden of Disease, and national sources due to differences in estimation methods, data availability, and modeling approaches. We have primarily used MMEIG estimates for consistency, but acknowledge that temporal comparisons are limited by these methodological differences. Data for 1975–1985 were unavailable, limiting our ability to assess trends in the earliest study decade.

Fourth, limitations of VOSviewer: The tool is optimized for up to 1,000 documents and lacks stemming functionality and geospatial mapping capabilities. Its reliance on network graphics without temporal analysis features necessitated a complementary manual analysis of trends.

Fifth, underreporting of indirect causes and community deaths: In SSA, particularly in underserved, rural, and conflict-affected regions, maternal deaths occurring outside health facilities are systematically undercounted in both vital registration and research studies. This leads to underestimation of true MMRs and potential misrepresentation of cause-of-death distributions.

Sixth, lack of quantitative meta-analysis: This bibliometric review did not statistically pool findings across studies, limiting our ability to generate summary effect estimates for risk factors or interventions.

Seventh, the most-cited publications analysis was subject to citation age bias: older publications have had more time to accumulate citations than recent ones. While we have included a citations-per-year metric in Supplementary Table S4 to partially adjust for this, the ranking remains inherently time-dependent. This limitation is recognized in bibliometric literature and should be considered when interpreting the influence of recent vs. historical research (69).

Finally, the heterogeneity of study designs, populations, and time periods limits direct cross-comparison of findings across the five decades studied.

### Policy implications and recommendations

These findings have direct implications for policy frameworks at global, regional, and national levels. The persistent mismatch between research output and maternal health outcomes highlights the need to accelerate progress toward SDG 3.1 (reducing global MMR to <70 deaths per 100,000 live births by 2030). Current trajectories suggest the majority of SSA countries will not achieve this target without transformative action. Our findings support the WHO maternal health roadmap's emphasis on strengthening health systems, addressing inequities, and improving data quality. They also align with the African Union's Campaign on Accelerated Reduction of Maternal Mortality in Africa (CARMMA), which prioritizes political commitment, resource mobilization, and health systems strengthening.

#### Evidence-based recommendations

1. Strengthen local research ecosystems: National governments should allocate dedicated budgets for maternal health research, targeting at least 2% of health budgets to research and development, as recommended by the WHO. This should include investments in research infrastructure, ethical review capacity, and data management systems.
2. Address funding inequities: International donors and philanthropic foundations should prioritize funding for under-researched thematic areas (socio-cultural determinants, health system governance, mental health, conflict settings) and underrepresented countries (Chad, South Sudan, the Central African Republic, and Sierra Leone). Funding mechanisms should require and resource equitable North–South partnerships with genuine local leadership.
3. Improve maternal mortality surveillance: Countries should strengthen Civil Registration and Vital Statistics (CRVS) systems, implement confidential maternal death reviews in all facilities, and develop community-based death notification systems to capture deaths outside facilities. The success of South Africa's National Committee on Confidential Enquiries into Maternal Deaths (NCCEMD) offers a replicable model.
4. Bridge research-policy gaps: Establish formal mechanisms for research translation, including policy briefs, evidence-to-action workshops, and embedded researchers in ministries of health. Research funders should require dissemination plans targeting policymakers and implementation actors, not just academic publications.
5. Promote South–South cooperation: Facilitate knowledge exchange among SSA countries through regional research networks, shared training programs, and collaborative multicountry studies. Countries that have made substantial progress (Cabo Verde, Mauritius, Seychelles, Rwanda, and Ethiopia) should share lessons with those lagging.
6. Address geopolitical vulnerabilities: The recent disruption of global health funding (e.g., changes in U.S. foreign aid policy) underscores the need for African ownership and diversified funding sources. Regional bodies (African Union, African Development Bank, and regional economic communities) should establish pooled funding mechanisms for maternal health research to buffer against external shocks.
7. Support non-English language dissemination: Journals and funders should encourage and resource translation of key findings into French, Portuguese, and local languages to reach policymakers and practitioners in Francophone and Lusophone Africa.

## Conclusion

Maternal mortality in SSA reflects broader systemic inequities. This study reveals a mismatch between research growth and maternal health improvements. While research output is increasing, its utility remains constrained by limited equity, translation, and ownership. A shift toward inclusive, context-driven research that informs national policies and prioritizes health equity is urgently needed. In addition, strengthening local research ecosystems and bridging research–policy gaps are imperative to make tangible progress.

## Data Availability

The original contributions presented in the study are included in the article/Supplementary Material, further inquiries can be directed to the corresponding author.
