# Supplementary material for: Fifty years of maternal mortality research in sub-Saharan Africa: a bibliometric analysis of trends, gaps, and opportunities for health equity
Source: Front Glob Womens Health. 2026 Jul 8;7:1717410. doi: 10.3389/fgwh.2026.1717410 (PMC13388389; doi:10.3389/fgwh.2026.1717410)
Supplement: Supplementary file 1 [file Supplementaryfile1.docx]

**List of Supplementary Materials**

***Supplementary File S1. Search Strategy for publications on maternal mortality in SSA, 1975 – 2024.***

To search for relevant literature on maternal mortality in SSA, the focus was to identify all relevant research on MM, and ensure a comprehensive and focused dataset for analysis, while at the same time minimizing any bias. Broad and inclusive search was conducted using all the terms entered into the Boolean operators. The Boolean operators, truncations and wildcards used included the following search strings: "maternal mortality” OR “postpartum hemorrhage*” OR “Traditional birth attendants” OR “Pregnancy complications”, with searches conducted within the Title-Abstract-Keyword field. Any article retrieved including (‘Postpartum hemorrhage’) AND (‘Bleeding’) AND (‘Hemorrhage’) AND (‘Risk factors’) AND (‘Determinants of health’) AND (‘Cause of death’) AND (‘ante- partum hemorrhage’) AND (‘Post-natal infections and sepsis’) AND (‘Pregnancy-induced hypertension’) AND (‘Peri-natal complication’) AND (‘Dystocia’) AND (‘Uterine rupture’) AND (‘Uterine prolapse’) AND (‘Unsafe abortion’) AND (‘Eclampsia’) AND (‘Pre-eclampsia’)) (Figure 1). The initial number of publications retrieved from Scopus (n = 75,637). The retrieved publications were in different languages representative of Sub-Saharan African countries represented (n = 46). This 50-year data extraction and analysis were believed to be sufficient to give an overview of maternal mortality research outputs from Sub-Saharan African.

**Suplementary Table S1: Estimates of Maternal Mortality Ratio in Sub-Saharan Africa, 1990 – 2023.**

| Country | Estimates of Maternal Mortality Ratio (maternal deaths per 100,000 live births) | | | | | | | |
| --- | --- | --- | --- | --- | --- | --- | --- | --- |
|  | 1990 | 1995 | 2000 | 2005 | 2010 | 2015 | 2020 | 2023 |
| Angola | 1400 | 1400 | 860 | 550 | 367 | 274 | 222 | 183 |
| Burundi | 1300 | 1300 | 874 | 713 | 608 | 514 | 494 | 392 |
| Benin | 600 | 520 | 469 | 509 | 598 | 591 | 523 | 518 |
| Burkina Faso | 770 | 680 | 506 | 417 | 357 | 295 | 264 | 242 |
| Botswana | 360 | 370 | 182 | 201 | 156 | 184 | 186 | 155 |
| Central African Republic | 1200 | 1200 | 1315 | 1158 | 1052 | 909 | 835 | 692 |
| Chad | 1700 | 1600 | 1366 | 1241 | 1303 | 1125 | 1063 | 748 |
| Côte d’Ivoire | 740 | 710 | 473 | 540 | 604 | 530 | 480 | 359 |
| Cameroon | 720 | 760 | 651 | 573 | 527 | 447 | 438 | 258 |
| Dem. Republic of the Congo | 1000 | 1100 | 668 | 635 | 601 | 578 | 547 | 427 |
| Congo | 670 | 650 | 660 | 488 | 389 | 360 | 282 | 241 |
| Comoros | 630 | 560 | 456 | 360 | 316 | 261 | 217 | 179 |
| Cabo Verde | 230 | 140 | 125 | 77 | 54 | 47 | 42 | 40 |
| Eritrea | 1700 | 1000 | 735 | 546 | 480 | 399 | 322 | 291 |
| Eswatini (formerly Swaziland) | 550 | 480 | 588 | 678 | 672 | 338 | 240 | 118 |
| Ethiopia | 1400 | 1200 | 953 | 880 | 635 | 399 | 267 | 195 |
| Equatorial Guinea | 1600 | 1300 | 427 | 258 | 211 | 201 | 212 | 174 |
| Gabon | 380 | 340 | 249 | 239 | 193 | 212 | 227 | 233 |
| The Gambia | 710 | 660 | 778 | 718 | 620 | 535 | 458 | 354 |
| Ghana | 760 | 650 | 499 | 390 | 337 | 286 | 263 | 234 |
| Guinea | 1100 | 1000 | 971 | 846 | 741 | 649 | 553 | 494 |
| Guinea-Bissau | 930 | 790 | 1300 | 977 | 795 | 713 | 725 | 505 |
| Kenya | 490 | 530 | 564 | 503 | 476 | 483 | 530 | 149 |
| Liberia | 1200 | 1600 | 777 | 676 | 634 | 686 | 652 | 628 |
| Lesotho | 720 | 630 | 545 | 563 | 1040 | 728 | 566 | 478 |
| Madagascar | 740 | 640 | 658 | 568 | 497 | 482 | 392 | 445 |
| Mali | 1100 | 1000 | 742 | 560 | 547 | 494 | 440 | 367 |
| Mozambique | 1300 | 1100 | 532 | 404 | 322 | 226 | 127 | 99 |
| Mauritania | 630 | 550 | 684 | 652 | 586 | 510 | 464 | 381 |
| Mauritius | 70 | 68 | 52 | 48 | 55 | 57 | 84 | 66 |
| Malawi | 1100 | 870 | 573 | 347 | 513 | 445 | 381 | 225 |
| Namibia | 320 | 280 | 450 | 419 | 482 | 299 | 215 | 139 |
| Niger | 1000 | 920 | 867 | 768 | 594 | 491 | 441 | 350 |
| Nigeria | 1200 | 1100 | 1148 | 1073 | 1123 | 1113 | 1047 | 993 |
| Rwanda | 1400 | 1400 | 1007 | 533 | 386 | 312 | 259 | 229 |
| Sao Tome and Principe | 410 | 360 | 179 | 160 | 160 | 139 | 146 | 75 |
| Senegal | 530 | 510 | 638 | 519 | 450 | 321 | 261 | 237 |
| Seychelles | - | - | 44 | 15 | 8 | 5 | 3 | 42 |
| Sierra Leone | 2300 | 2400 | 1682 | 1327 | 837 | 588 | 443 | 354 |
| South Africa | 150 | 140 | 173 | 221 | 219 | 141 | 127 | 118 |
| South Sudan | 1800 | 1500 | 1687 | 1332 | 1060 | 1225 | 1223 | 692 |
| Togo | 660 | 660 | 479 | 494 | 530 | 441 | 399 | 349 |
| United Republic of Tanzania | 910 | 890 | 760 | 559 | 486 | 330 | 238 | 276 |
| Uganda | 780 | 740 | 461 | 435 | 372 | 319 | 284 | 170 |
| Zambia | 580 | 630 | 419 | 309 | 268 | 166 | 135 | 85 |
| Zimbabwe | 520 | 550 | 388 | 533 | 618 | 408 | 357 | 358 |

*Data for 2000 – 2020 were retrieved from the report of the Internationally comparable MMR estimates by the Maternal Mortality Inter-Agency Group (MMEIG): WHO, UNICEF, UNFPA, World Bank Group and the United Nations Population Division. Available at:* <https://www.who.int/publications/i/item/9789240068759>*. Values for 1990 and 1995 were retrieved from Estimates from* *Trends in maternal mortality: 1990 to 2013. Estimates by WHO, UNICEF, UNFPA, The World Bank and the United Nations Population Division (WHO, 2014). Data for earlier years (1975 – 1985) were unavailable.*

***Supplementary List 2. References 1 - 16. List of references associated with Table 1.***

- - - 1. Say, L., Chou, D., Gemmill, A., Tunçalp, Ö., Moller, A. B., Daniels, J., Gülmezoglu, A. M., Temmerman, M., & Alkema, L. (2014). Global causes of maternal death: a WHO systematic analysis. *The Lancet. Global health*, 2(6), e323–e333. <https://doi.org/10.1016/S2214-109X(14)70227-X>.
      2. Pattinson, R. C., Buchmann, E., Mantel, G., Schoon, M., & Rees, H. (2003). Can enquiries into severe acute maternal morbidity act as a surrogate for maternal death enquiries?. BJOG : an international journal of obstetrics and gynaecology, 110(10), 889–893.
      3. Ujah, I. A., Aisien, O. A., Mutihir, J. T., Vanderjagt, D. J., Glew, R. H., & Uguru, V. E. (2005). Factors contributing to maternal mortality in north-central Nigeria: a seventeen-year review. *African journal of reproductive health*, 9(3), 27–40.
      4. Berhan, Y., & Berhan, A. (2014). Causes of maternal mortality in Ethiopia: a significant decline in abortion related death. *Ethiopian journal of health sciences*, 24 Suppl(0 Suppl), 15–28. <https://doi.org/10.4314/ejhs.v24i0.3s>.
      5. Mekonnen, W., Hailemariam, D., Gebremariam, A. (2018). Causes of maternal death in Ethiopia between 1990 and 2016: Systematic review with meta-analysis. *Ethiopian Journal of Health Development*, 2018, 32(4), pp. 225–242.
      6. Ali, A. A., Khojali, A., Okud, A., Adam, G. K., & Adam, I. (2011). Maternal near-miss in a rural hospital in Sudan. *BMC pregnancy and childbirth*, 11, 48. <https://doi.org/10.1186/1471-2393-11-48>.
      7. Gelaye, A. A., Taye, K. N., & Mekonen, T. (2014). Magnitude and risk factors of abortion among regular female students in Wolaita Sodo University, Ethiopia. *BMC women's health*, 14, 50. <https://doi.org/10.1186/1472-6874-14-50>.
      8. Adeoye, I. A., Onayade, A. A., & Fatusi, A. O. (2013). Incidence, determinants and perinatal outcomes of near miss maternal morbidity in Ile-Ife Nigeria: a prospective case control study. *BMC pregnancy and childbirth*, 13, 93. <https://doi.org/10.1186/1471-2393-13-93>.
      9. Ntoimo, L. F., Okonofua, F. E., Ogu, R. N., Galadanci, H. S., Gana, M., Okike, O. N., Agholor, K. N., Abdus-Salam, R. A., Durodola, A., Abe, E., & Randawa, A. J. (2018). Prevalence and risk factors for maternal mortality in referral hospitals in Nigeria: a multicenter study. *International journal of women's health*, 10, 69–76. <https://doi.org/10.2147/IJWH.S151784>.
      10. Sepanlou, S. G., Rezaei Aliabadi, H., Malekzadeh, R., Naghavi, M., & GBD Maternal Middle East Collaborators (2022). Levels and Trends of Maternal Mortality and Morbidity by Cause in North Africa and Middle East, 1990 to 2019: An Analysis for the Global Burden of Disease Study 2019. *Archives of Iranian medicine*, 25(10), 666–675. <https://doi.org/10.34172/aim.2022.105>.
      11. Tessema, G. A., Laurence, C. O., Melaku, Y. A., Misganaw, A., Woldie, S. A., Hiruye, A., Amare, A. T., Lakew, Y., Zeleke, B. M., & Deribew, A. (2017). Trends and causes of maternal mortality in Ethiopia during 1990-2013: findings from the Global Burden of Diseases study 2013. *BMC public health*, 17(1), 160. <https://doi.org/10.1186/s12889-017-4071-8>.
      12. Bouvier-Colle, M. H., Ouedraogo, C., Dumont, A., Vangeenderhuysen, C., Salanave, B., Decam, C., & MOMA group (2001). Maternal mortality in West Africa. Rates, causes and substandard care from a prospective survey. *Acta obstetricia et gynecologica Scandinavica*, 80(2), 113–119.
      13. Saucedo, M., Deneux-Tharaux, C., & pour le comité national d’experts sur les mort maternelles (CNEMM) (2024). Mortalité maternelle en France, 2016–2018, fréquence, causes et profil des femmes [Maternal mortality in France, 2016-2018, frequency, causes and women's profile]. *Gynecologie, obstetrique, fertilite & senologie*, 52(4), 185–200. <https://doi.org/10.1016/j.gofs.2024.02.020>.
      14. Illah, E., Mbaruku, G., Masanja, H., & Kahn, K. (2013). Causes and risk factors for maternal mortality in rural Tanzania--case of Rufiji Health and Demographic Surveillance Site (HDSS). *African journal of reproductive health*, 17(3), 119–130.
      15. Manyeh, A. K., Nathan, R., & Nelson, G. (2018). Maternal mortality in Ifakara Health and Demographic Surveillance System: Spatial patterns, trends and risk factors, 2006 - 2010. *PloS one*, 13(10), e0205370. <https://doi.org/10.1371/journal.pone.0205370>.
      16. Rogo, K. O., Oucho, J. & Mwalali, P. (2006). Maternal Mortality, Chapter 16. In: Jamison, D. T., Feachem, R. G., Makgoba, M. W., Bos, E. R., Baingana, F. K., Hofman, K. J., & Rogo, K. O. (Eds.). *Disease and Mortality in Sub-Saharan Africa*, 2nd ed. The World Bank, Washington, USA. Available at: https://www.ncbi.nlm.nih.gov/books/NBK2279/pdf/Bookshelf_NBK2279.pdf. Accessed 12 March 2025.
      17. Page, M. J., Moher, D., Bossuyt, P. M., Boutron, I., Hoffmann, T. C., Mulrow, C. D., ... & McKenzie, J. E. (2021). PRISMA 2020 explanation and elaboration: updated guidance and exemplars for reporting systematic reviews. BMJ 2021;372:n160. http://dx.doi.org/10.1136/bmj.n160.
      18. Kyomuhendo, G. B. (2003). Low Use of Rural Maternity Services in Uganda: Impact of Women's Status, Traditional Beliefs and Limited Resources. *Reproductive Health Matters*, 11(21), 16–26. <https://doi.org/10.1016/S0968-8080(03)02176-1>.
      19. Ujah, I. A., Aisien, O. A., Mutihir, J. T., Vanderjagt, D. J., Glew, R. H., & Uguru, V. E. (2005). Factors contributing to maternal mortality in north-central Nigeria: a seventeen-year review. *African journal of reproductive health*, 9(3), 27–40.
      20. Regassa N. (2011). Antenatal and postnatal care service utilization in southern Ethiopia: a population-based study. African health sciences, 11(3), 390–397.
      21. Abdella, A. (2010). Maternal mortality trend in Ethiopia. *Ethiopian Journal of Health Development*, 24(Special Issue 1), 115 – 122. DOI:10.4314/ejhd.v24i1.62953.
      22. Berhan, Y., & Berhan, A. (2014). Causes of maternal mortality in Ethiopia: a significant decline in abortion related death. *Ethiopian journal of health sciences*, 24 Suppl(0 Suppl), 15–28. <https://doi.org/10.4314/ejhs.v24i0.3s>.
      23. Greenwood, A. M., Greenwood, B. M., Bradley, A. K., Williams, K., Shenton, F. C., Tulloch, S., Byass, P., & Oldfield, F. S. (1987). A prospective survey of the outcome of pregnancy in a rural area of the Gambia. *Bulletin of the World Health Organization*, 65(5), 635–643.
      24. Oladapo, O. T., Sule-Odu, A. O., Olatunji, A. O., & Daniel, O. J. (2005). "Near-miss" obstetric events and maternal deaths in Sagamu, Nigeria: a retrospective study. *Reproductive health*, 2, 9. <https://doi.org/10.1186/1742-4755-2-9>.
      25. Wanjira, C., Mwangi, M., Mathenge, E., Mbugua, G., & Ng'ang'a, Z. (2011). Delivery practices and associated factors among mothers seeking child welfare services in selected health facilities in Nyandarua South District, Kenya. *BMC public health*, 11, 1-9.
      26. Pattinson, R. C., Buchmann, E., Mantel, G., Schoon, M., & Rees, H. (2003). Can enquiries into severe acute maternal morbidity act as a surrogate for maternal death enquiries?. BJOG : an international journal of obstetrics and gynaecology, 110(10), 889–893.
      27. Fasubaa, O. B., Ezechi, O. C., Orji, E. O., Ogunniyi, S. O., Akindele, S. T., Loto, O. M., & Okogbo, F. O. (2002). Delivery of the impacted head of the fetus at caesarean section after prolonged obstructed labour: a randomised comparative study of two methods. *Journal of obstetrics and gynaecology* : the journal of the Institute of Obstetrics and Gynaecology, 22(4), 375–378. <https://doi.org/10.1080/01443610220141290>.
      28. Paruk, F., & Moodley, J. (2000). Maternal and neonatal outcome in early- and late-onset pre-eclampsia. *Seminars in neonatology* : SN, 5(3), 197–207. <https://doi.org/10.1053/siny.2000.0023>.
      29. Bhagwanjee, S., Paruk, F., Moodley, J., & Muckart, D. J. (2000). Intensive care unit morbidity and mortality from eclampsia: an evaluation of the Acute Physiology and Chronic Health Evaluation II score and the Glasgow Coma Scale score. *Critical care medicine*, 28(1), 120-124.
      30. Moodley, J., Pattinson, R. C., Fawcus, S., Schoon, M. G., Moran, N., Shweni, P. M., & National Committee on Confidential Enquiries into Maternal Deaths in South Africa (2014). The Confidential Enquiry into Maternal Deaths in South Africa: a case study. *BJOG*: an international journal of obstetrics and gynaecology, 121 Suppl 4, 53–60. <https://doi.org/10.1111/1471-0528.12869>.
      31. Musarandega R, Nyakura M, Machekano R, Pattinson R, Munjanja SP. Causes of maternal mortality in Sub-Saharan Africa: A systematic review of studies published from 2015 to 2020. J Glob Health. 2021 Oct 9;11:04048. doi: 10.7189/jogh.11.04048.
      32. Ganle, J. K. (2015). Why Muslim women in Northern Ghana do not use skilled maternal healthcare services at health facilities: a qualitative study. *BMC Int Health Hum Rights* 15, 10. <https://doi.org/10.1186/s12914-015-0048-9>.

**Suplementary Table S3: Identified countries contributing to peer-reviewed discussions on maternal mortality in sub-Saharan Africa, 1975 – 2024, (n = 46)**

| **Identified sub-Saharan Africa countries** | **Number of peer reviewed documents on maternal mortality available** |
| --- | --- |
| Nigeria | 534 |
| South Africa | 385 |
| Ethiopia | 343 |
| Ghana | 94 |
| Egypt | 83 |
| Kenya | 74 |
| Uganda | 60 |
| Tunisia | 58 |
| Tanzania | 54 |
| Morocco | 46 |
| Zimbabwe | 44 |
| Burkina Faso | 36 |
| Senegal | 27 |
| Cameroon | 26 |
| Sudan | 26 |
| Malawi | 23 |
| Gabon | 18 |
| Zambia | 17 |
| Congo | 16 |
| Botswana | 14 |
| Democratic Republic Congo | 12 |
| Cote d'Ivoire | 11 |
| Niger | 9 |
| Sierra Leone | 9 |
| Benin | 8 |
| Gambia | 8 |
| Somalia | 8 |
| Algeria | 7 |
| Guinea | 7 |
| Madagascar | 7 |
| Mozambique | 6 |
| Swaziland | 6 |
| Togo | 6 |
| Rwanda | 4 |
| Central African Republic | 3 |
| Eritrea | 3 |
| Liberia | 3 |
| Mali | 3 |
| Mauritania | 3 |
| Mauritius | 3 |
| Burundi | 2 |
| Chad | 2 |
| Libyan Arab Jamahiriya | 2 |
| Guinea-Bissau | 1 |
| Lesotho | 1 |
| Namibia | 1 |

**Suplementary Table S4: Top 15 most influential publications on maternal mortality in Sub-Saharan Africa based on total citations**

| Publication | Title | Summary of research | Journal | Scopus citations | NIH citations |
| --- | --- | --- | --- | --- | --- |
| Kyomuhendo, (2003) | Low use of rural maternity services in Uganda: Impact of women's status, traditional beliefs and limited resources | The paper reports a study from rural district of western Uganda, to understand why women continue to choose high risk options when faced with complications of pregnancy or delivery, leading to severe morbidity and mortality. Traditional birthing practices, beliefs that pregnancy is a test of endurance, and maternal deaths were perceived as normal event. Primary health units and the referral hospital are perceived as last resorts. | RHM | 198 | 497 |
| Ujah, et al., (2005) | Factors contributing to maternal mortality in north-central Nigeria: a seventeen-year review | Maternal mortality ratio in Nigeria is one of the highest in the world. The study was conducted in north-central Nigeria to determine the magnitude, trends, causes and characteristics of maternal deaths before and after the launch of the Safe Motherhood Initiative in Nigeria. Socio-biological variables (age, booking status, educational level, parity, ethnic group, marital status, among others) were causes of maternal deaths. The major direct causes of MM were haemorrhage (34.6%), sepsis (28.3%), eclampsia (23.6%) and unsafe abortion (9.6%). | AJRH | 98 | 308 |
| Regassa (2011) | Antenatal and postnatal care service utilization in southern Ethiopia: a population-based study | The study examined prevalence and factors associated with antenatal Care (ANC) and Postnatal Care (PNC) service utilizations. It revealed that the level of ANC and PNC service utilizations is 77.4 % and 37.2% respectively. Women who are literate, have exposure to media, and women with low parity are more likely to use both ANC and PNC services. ANC utilization was generally better than for PNC. Promoting women's education and behavioural change communication at rural level is beneficial. | AHS | 123 | 267 |
| Abdella (2010) | Maternal mortality trend in Ethiopia | Study aimed at reviewing the maternal mortality trend and causes in Ethiopia. There was decreasing maternal mortality though the current maternal mortality ratio was still high, and the proportion of maternal deaths due to the each of the five major causes varies with time. There is no grossly notable change in proportion of deaths due to the main causes. There is an urgent need to improve the quality of maternal health services. | EJHD | 85 | 233 |
| Berhan & Berhan, (2014) | Causes of Maternal Mortality in Ethiopia: A Significant Decline in Abortion Related Death | Using a computer-based literature search in databases maternal mortality ratio (MMR) for Ethiopia was reviewed for 30 years. Data from international databases were inconsistent and there has been no significant change in maternal mortality over the last three decades. The MDGs and HSDP envisaged significant improvement in maternal health, but targets were not met. | EJHS | 97 | 223 |
| Greenwood et al., (1987) | A prospective survey of the outcome of pregnancy in a rural area of the Gambia | Outcome of pregnancy was studied among 672 women in rural Gambia with limited healthcare resources. MM was high and post-partum haemorrhage and sepsis were main causes of deaths. Under 20 and over 40 years have poor pregnancy outcomes, hence these groups need encouragement to deliver in healthcare centres and hospitals. | BWHO | 108 | 207 |
| Oladapo et al., (2005) | "Near-miss" obstetric events and maternal deaths in Sagamu, Nigeria: A retrospective study | In determining the frequency of severe acute maternal morbidity (SAMM), a three-year Nigerian based study was conducted. Study revealed the following causes/ complications of maternal deaths: hypertensive disorders in pregnancy and haemorrhage (50%), uterine rupture (37.5%) and infection (28.6%) and 88.6% were unbooked for antenatal care and hospital delivery. | RH | 82 | 201 |
| Wanjira et al., (2011) | Delivery practices and associated factors among mothers seeking child welfare services in selected health facilities in Nyandarua South District, Kenya | The hospital-based study aimed at establishing delivery practices and associated factors in mothers in Nyandarua South district, Kenya. Among 1170 deliveries, 51.8% were conducted by unskilled birth attendants including 38.6% done by neighbours and/or relatives, 1.5% by Traditional Birth Attendants and 11.7% were self-administered. Identified risk factors for unsafe delivery practice were poor education, > three deliveries in a lifetime, and mothers with perceived similarity in delivery attendance among skilled and unskilled delivery attendants. | BMC-PH | 56 | 165 |
| Pattinson et al., (2003) | Can enquiries into severe acute maternal morbidity act as a surrogate for maternal death enquiries? | Using SAMM as a proxy for maternal deaths, patterns and risk factor for maternal diseases were evaluated in rural and urban South Africa. SAMM’s complications were hypertension (27.2%), postpartum haemorrhage (18.0%), antepartum haemorrhage (12.8%) and abortion (11.3%), and causes of maternal death were non-pregnancy related sepsis (26.6%), complications of hypertension (23.4%), pre-existing medical disease (14.1%) and abortion (10.9%). | BJOG | 88 | 160 |
| Fasubaa et al., (2002) | Delivery of the impacted head of the fetus at caesarean section after prolonged obstructed labour: A randomised comparative study of two methods | The study compared morbidity and mortality associated with two delivery methods (push and pull) in cases of impacted foetal head at caesarean section for obstructed labour in Ile-Ife, Nigeria. Patients in the push group had higher rates of maternal morbidity (longer operation time, more blood loss, extension of uterine incision, endometritis, longer hospital stay and higher hospital bills) than the pull. | JOG | 77 | 146 |
| Paruk & Moodley (2000) | Maternal and neonatal outcome in early- and late-onset pre-eclampsia | The study reviewed and compared concepts of care in specialized units, expectant management of pre-eclampsia, conservative management of the HELLP syndrome (haemolysis, elevated liver enzymes, low platelets), usage of magnesium sulphate and improved feto-maternal surveillance in early-onset pre-eclampsia and late-onset pre-eclampsia based on the nature and predilection for multi-organ involvement in maternal and foetal death. These factors also influence maternal and neonatal outcome. | SN | 57 | 130 |
| Bhagwanjee et al., (2000) | Intensive care unit morbidity and mortality from eclampsia: An evaluation of the Acute Physiology and Chronic Health Evaluation II score and the Glasgow Coma Scale score | The study determined the maternal morbidity and mortality in patients with eclampsia admitted to an intensive care unit (ICU), and to establish the efficacy of the Acute Physiology and Chronic Health Evaluation (APACHE) II score. APACHE II score was higher in non-survivors, mean number of organ failures was higher in non-survivors compared with survivors. | CCM | 66 | 128 |
| Moodley et al., (2014) | The Confidential Enquiry into Maternal Deaths in South Africa: a case study | The Confidential Enquiry into Maternal Deaths (CEMD) study in South Africa was reviewed after 15 years. Earlier, institutional maternal mortality ratio of 176.2 per 100 000 live births in the 2008 - 2010 decreased to 146.7 in the 2011/2012 period. CEMD process was maintained and strengthened, and performed optimally at national and district levels to identify deficiencies within the health system, generate reports and also provide early warning about alarming trends that increase MM. | BJOG | 84 | 126 |
| Musarandega et al., (2021) | Causes of maternal mortality in Sub-Saharan Africa: A systematic review of studies published from 2015 to 2020 | A systematic review was conducted to identify the leading causes of maternal deaths using the international classification of disease – 10th revision, for maternal mortality (ICD-MM) and multiple databases. Results were compared the results with WHO and Global Burden of Disease (GDB) estimates. The leading causes of MM were obstetric haemorrhage (28.8%), hypertensive disorders in pregnancy (22.1%), non-obstetric complications (18.8%) and pregnancy-related infections (11.5%). | JGH | 64 | 124 |
| Ganle (2015) | Why Muslim women in Northern Ghana do not use skilled maternal healthcare services at health facilities: A qualitative study Healthcare availability and accessibility | Muslim women sub-population in Ghana has very low rate of skilled maternal health services accessibility and utilisation and no study has explored maternity needs and care experiences of Muslim women. Using qualitative research and thematic analysis, the study was conducted with 94 Muslim women in three communities. Muslim women do want to receive skilled care in a health facility, conditioned by a religious obligation to maintain bodily sanctity through modest dressing and the avoidance of unlawful bodily exposure or contact by male or alien caregivers. Other barriers include lack of privacy, healthcare providers’ insensitivity and lack of knowledge about Muslim women’s religious and cultural practices among others, Suiting intervention must be implemented. | BMC-IHHR | 51 | 110 |

*AHS = African Health Sciences; AJRH = African Journal of Reproductive Health; BJOG = BJOG: An International Journal of Obstetrics and Gynaecology; BMC-IHHR = BMC International Health and Human Rights (now merged with BMC Public Health); BMC-PH = BMC Public Health; B-WHO = Bulletin of the World Health Organization; CCM = Critical Care Medicine; EJHD = Ethiopian Journal of Health Development; EJHS = Ethiopian Journal of Health Sciences; JGH = Journal of Global Health; JOG = Journal of Obstetrics and Gynaecology; NIH = National Institutes of Health; RH = Reproductive Health; RHM = Reproductive Health Matters; SN = Seminars in Neonatology.*

**Suplementary Table S5: The most influential journals for publishing research on maternal mortality in Sub-Saharan Africa based on evaluated publications**

| Journal name | Journal Impact Factor (Clarivate) 2023 | CiteScore (Scopus) 2023 | Journal h-index | Total articles published on maternal mortality, SSA* | SNIP |
| --- | --- | --- | --- | --- | --- |
| *International Journal of Gynecology & Obstetrics* | 2.6 | 5.8 | 119 | 166 | 1.109 |
| *BMC Pregnancy and Childbirth* | 2.8 | 4.1 | 114 | 124 | 1.276 |
| *South African Medical Journal (SAMJ)* | 1.5 | 3.0 | 65 | 94 | 0.449 |
| *PLoS ONE* | 2.9 | 6.2 | 435 | 80 | 1.084 |
| *Pan African Medical Journal* | 0.9 | 1.8 | 49 | 79 | 0.460 |
| *Journal of Obstetrics and Gynaecology* | 0.9 | 2.4 | 57 | 76 | 0.544 |
| *African Journal of Reproductive Health* | 0.7 | 1.2 | 49 | 47 | 0.413 |
| *East African Medical Journal* | 0.026 | NA | 45 | 44 | 0.01 |
| *Reproductive Health* | 3.6 | 6.0 | 76 | 39 | 1.496 |
| *International Journal of Women’s Health* | 2.5 | 3.7 | 64 | 38 | 1.134 |
| *Tropical Doctor* | 0.5 | 1.2 | 39 | 36 | 0.361 |
| *Journal De Gynecologie Obstetrique Et Biologie De La Reproduction (Journal of Gynecology Obstetrics and Human Reproduction)* | 1.7 | 3.7 | 44 | 33 | 0.783 |
| *BMC Women’s Health* | 2.4 | 3.4 | 64 | 30 | 1.100 |
| *Revue Francaise De Gynecologie Et D Obstetrique#* | NA | NA | 5 | 29 | NA |
| *African Health Sciences* | 0.8 | 2.3 | 58 | 27 | 0.489 |
| *South African Journal of Obstetrics and Gynaecology* | 0.4 | 0.4 | 10 | 25 | 0.064 |
| *Nigerian Journal of Medicine* | NA | NA | 25 | 21 | NA |
| *Nigerian Journal of Clinical Practice* | 0.7 | 1.4 | 34 | 20 | 0.452 |
| *BMC Public Health* | 3.5 | 6.5 | 197 | 19 | 1.386 |
| *BMC Health Services Research* | 2.7 | 4.4 | 146 | 18 | 1.177 |
| *BJOG: An International Journal of Obstetrics and Gynaecology* | 4.7 | 10.9 | 188 | 16 | 1.976 |

*Source Normalized Impact per Paper (SNIP). Other influential journals with number of published articles on maternal mortality but which are not listed in the table above included the Central African journal of medicine (13), the Ethiopian Medical journal (12), the Ethiopian Journal of Reproductive Health (11), the Ethiopian Journal of Health Sciences (11), and Obstetrics and Gynaecology Forum (10). *Note that the metrics on total count of published articles are changing as new articles are added to the journals (also see Supplementary Table 3). #Details not found. The available publication range spread from 1919 – 1999.*

**Supplementary Table S6: Identified Funding Sources funding peer-reviewed research on maternal mortality in sub-Saharan Africa, 1975 – 2024, (n = 159)**

| *Funding Sources* | *Number of peer reviewed documents on maternal mortality funded* |
| --- | --- |
| *Carnegie Corporation of New York* | *19* |
| *South African Medical Research Council* | *14* |
| *Bill and Melinda Gates Foundation* | *13* |
| *University of Gondar* | *13* |
| *World Health Organization* | *13* |
| *National Institutes of Health* | *12* |
| *Styrelsen för Internationellt Utvecklingssamarbete* | *12* |
| *Jimma University* | *10* |
| *Medical Research Council* | *10* |
| *Fogarty International Center* | *9* |
| *National Research Foundation* | *9* |
| *Addis Ababa University* | *8* |
| *United States Agency for International Development* | *8* |
| *Wellcome Trust* | *8* |
| *United Nations Population Fund* | *7* |
| *Department for International Development, UK Government* | *6* |
| *Haramaya University* | *6* |
| *International Development Research Centre* | *6* |
| *Inyuvesi Yakwazulu-Natali* | *6* |
| *UNICEF* | *6* |
| *University of Pretoria* | *6* |
| *African Population and Health Research Center* | *5* |
| *Centers for Disease Control and Prevention* | *5* |
| *Hawassa University* | *5* |
| *Mekelle University* | *5* |
| *U.S. Department of Health and Human Services* | *5* |
| *U.S. President’s Emergency Plan for AIDS Relief* | *5* |
| *University of Venda* | *5* |
| *University of the Witwatersrand, Johannesburg* | *5* |
| *William and Flora Hewlett Foundation* | *5* |
| *European Commission* | *4* |
| *GlaxoSmithKline* | *4* |
| *Government of the United Kingdom* | *4* |
| *Johns Hopkins University* | *4* |
| *Ministry of Health* | *4* |
| *National Department of Health* | *4* |
| *National Institute of Allergy and Infectious Diseases* | *4* |
| *National Institute of Mental Health* | *4* |
| *University of South Africa* | *4* |
| *African Academy of Sciences* | *3* |
| *Afya Bora Consortium* | *3* |
| *Ambo University* | *3* |
| *Columbia University* | *3* |
| *Department for International Development* | *3* |
| *Department of Science and Technology, Ministry of Science and Technology, India* | *3* |
| *HRP Alliance for Research Capacity Strengthening* | *3* |
| *Health Resources and Services Administration* | *3* |
| *Makerere University* | *3* |
| *New Partnership for Africa's Development* | *3* |
| *Office of AIDS Research* | *3* |
| *Office of the Director* | *3* |
| *Pennsylvania College of Health Sciences* | *3* |
| *University of Washington* | *3* |
| *Bill and Melinda Gates Institute for Population and Reproductive Health* | *2* |
| *Council for the Development of Social Science Research in Africa* | *2* |
| *Debre Markos University* | *2* |
| *Debre Tabor University* | *2* |
| *Eunice Kennedy Shriver National Institute of Child Health and Human Development* | *2* |
| *International Association for Suicide Prevention* | *2* |
| *Ministerio de Sanidad, Consumo y Bienestar Social* | *2* |
| *Ministry of Health of the People's Republic of China* | *2* |
| *Nederlandse Organisatie voor Wetenschappelijk Onderzoek* | *2* |
| *North-West University* | *2* |
| *Population Council* | *2* |
| *Rockefeller Foundation* | *2* |
| *Salale University* | *2* |
| *Science and Technology Development Fund* | *2* |
| *United Nations Fund for Population Activities* | *2* |
| *University of Cape Town* | *2* |
| *University of Ghana* | *2* |
| *University of Ibadan* | *2* |
| *African Union* | *1* |
| *African Union Commission* | *1* |
| *Agence Nationale de la Recherche* | *1* |
| *Ain Shams University* | *1* |
| *Aksum University* | *1* |
| *American University* | *1* |
| *American University of Beirut* | *1* |
| *Amhara Regional Health Bureau* | *1* |
| *Bahir Dar University* | *1* |
| *Bank of Canada* | *1* |
| *Belgisch Ontwikkelingsagentschap* | *1* |
| *Bundesministerium für Gesundheit* | *1* |
| *Bundesministerium für Wirtschaftliche Zusammenarbeit und Entwicklung* | *1* |
| *Busitema University* | *1* |
| *Centers for Disease Control and Prevention Foundation* | *1* |
| *College of Medicine and Health Sciences, University of Gondar* | *1* |
| *Comic Relief* | *1* |
| *Conselho Nacional de Desenvolvimento Científico e Tecnológico* | *1* |
| *Copenhagen Trial Unit, Centre for Clinical Intervention Research* | *1* |
| *Covenant University* | *1* |
| *Danish International Development Agency* | *1* |
| *Department of Foreign Affairs and Trade* | *1* |
| *Department of Health and Social Care* | *1* |
| *Department of Health, Western Cape Government* | *1* |
| *Department of Human Services, State Government of Victoria* | *1* |
| *Department of Obstetrics and Gynecology, University of Wisconsin-Madison* | *1* |
| *Department of Science and Technology, Republic of South Africa* | *1* |
| *Dilla University* | *1* |
| *Discovery Eye Foundation* | *1* |
| *Durban University of Technology* | *1* |
| *Ethiopian Public Health Association* | *1* |
| *Fetal Medicine Foundation* | *1* |
| *Fondation Générale de Santé* | *1* |
| *Ford Foundation* | *1* |
| *Foundation for the Author of National Excellent Doctoral Dissertation of the People's Republic of China* | *1* |
| *Fundação para a Ciência e a Tecnologia* | *1* |
| *Gentofte Hospital* | *1* |
| *Gilead Sciences* | *1* |
| *Global Affairs Canada* | *1* |
| *Global Fund to Fight AIDS, Tuberculosis and Malaria* | *1* |
| *Government of the Republic of Kenya* | *1* |
| *Harvard T.H. Chan School of Public Health* | *1* |
| *Hebrew University of Jerusalem* | *1* |
| *Hôpitaux Universitaires de Genève* | *1* |
| *Institute for Healthcare Improvement* | *1* |
| *Institute of International Education* | *1* |
| *Instituto de Salud Carlos III* | *1* |
| *Intensive Care Foundation* | *1* |
| *International AIDS Society* | *1* |
| *International AIDS Vaccine Initiative* | *1* |
| *John D. and Catherine T. MacArthur Foundation* | *1* |
| *Johns Hopkins Bloomberg School of Public Health* | *1* |
| *Kansas Housing Resources Corporation* | *1* |
| *LIFE programme* | *1* |
| *London School of Hygiene and Tropical Medicine* | *1* |
| *Mailman School of Public Health, Columbia University* | *1* |
| *Maternal and Child Health Bureau* | *1* |
| *Medical Research Foundation* | *1* |
| *Meso Scale Diagnostics* | *1* |
| *Ministry of Education, Ethiopia* | *1* |
| *Ministry of Environmental Protection* | *1* |
| *Ministry of Higher Education and Scientific Research* | *1* |
| *Ministry of Science and Technology* | *1* |
| *Ministério da Educação e Ciência* | *1* |
| *Minnesota Department of Health* | *1* |
| *Muhimbili University of Health and Allied Sciences* | *1* |
| *National Commission for Science, Technology and Innovation* | *1* |
| *National Institute for Health Research* | *1* |
| *National Institute for Health and Care Research* | *1* |
| *National Institute of Child Health and Human Development* | *1* |
| *National Institute of Neurological Disorders and Stroke* | *1* |
| *National Institute on Minority Health and Health Disparities* | *1* |
| *National Research Foundation of Korea* | *1* |
| *Niche* | *1* |
| *Office of the President, University of California* | *1* |
| *Pan African Thoracic Society* | *1* |
| *Polska Akademia Nauk* | *1* |
| *Public Health Institute* | *1* |
| *Saint Paul's Hospital Millennium Medical College* | *1* |
| *Servier* | *1* |
| *Seventh Framework Programme* | *1* |
| *Shandong Province* | *1* |
| *Simmons College* | *1* |
| *Spirit AeroSystems* | *1* |
| *St. Joseph Health Ministries* | *1* |
| *Swedish Foundation for International Cooperation in Research and Higher Education* | *1* |
| *Tertiary Education Trust Fund* | *1* |
| *UNC Department of Obstetrics and Gynecology* | *1* |

| 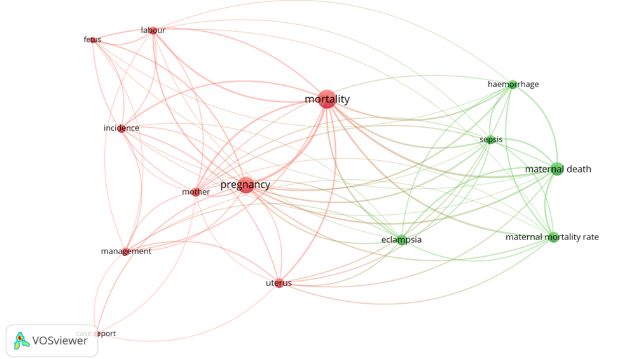 | 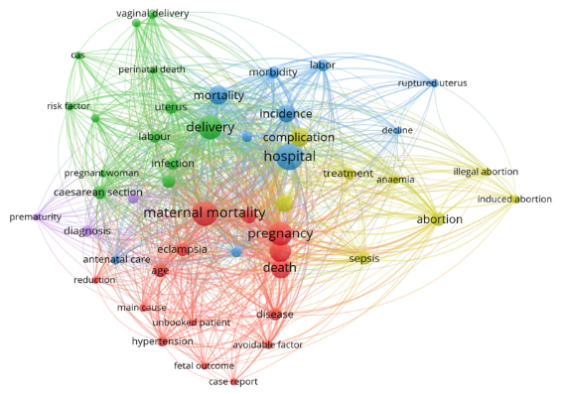 | | 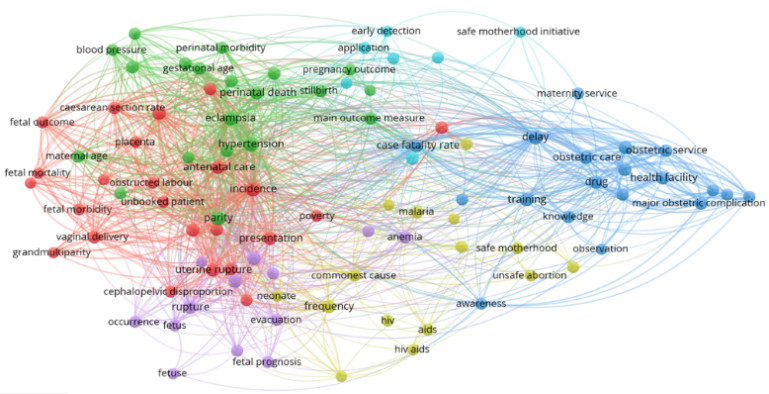 |
| --- | --- | --- | --- |
| 1. 1975 – 1984 | 1. 1985 – 1994 | | 1. 1995 – 2004 |
| 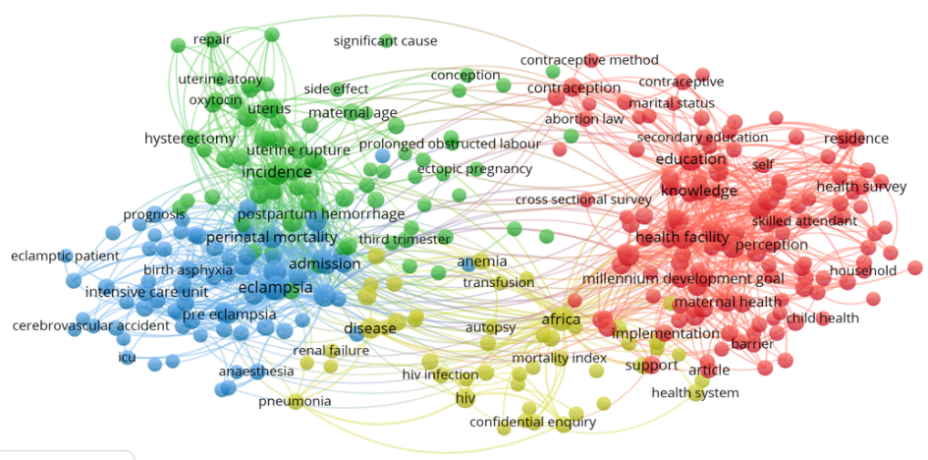 | | 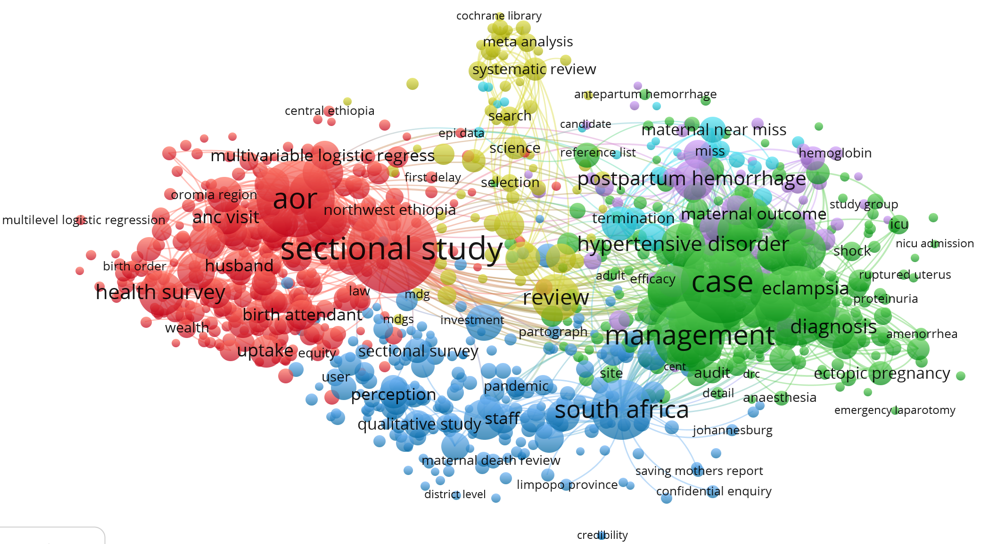 | |
| 1. 2005 – 2014 | | 1. 2015 – August 2024 | |

***Suplementary Figure S1. Decade-wise Network visualization of key terms in peer-reviewed publications on maternal mortality research in Sub-Saharan Africa, 1975 - 2024.***

***Supplementary Figure S2. Two temporal block-wise (1975-2014 & 2015-2024) Network visualization of key terms in peer-reviewed publications on maternal mortality research in sub-Saharan Africa, 1975 - 2024.*** **Enlarged representation of the pictures in Supplementary figure 2.*


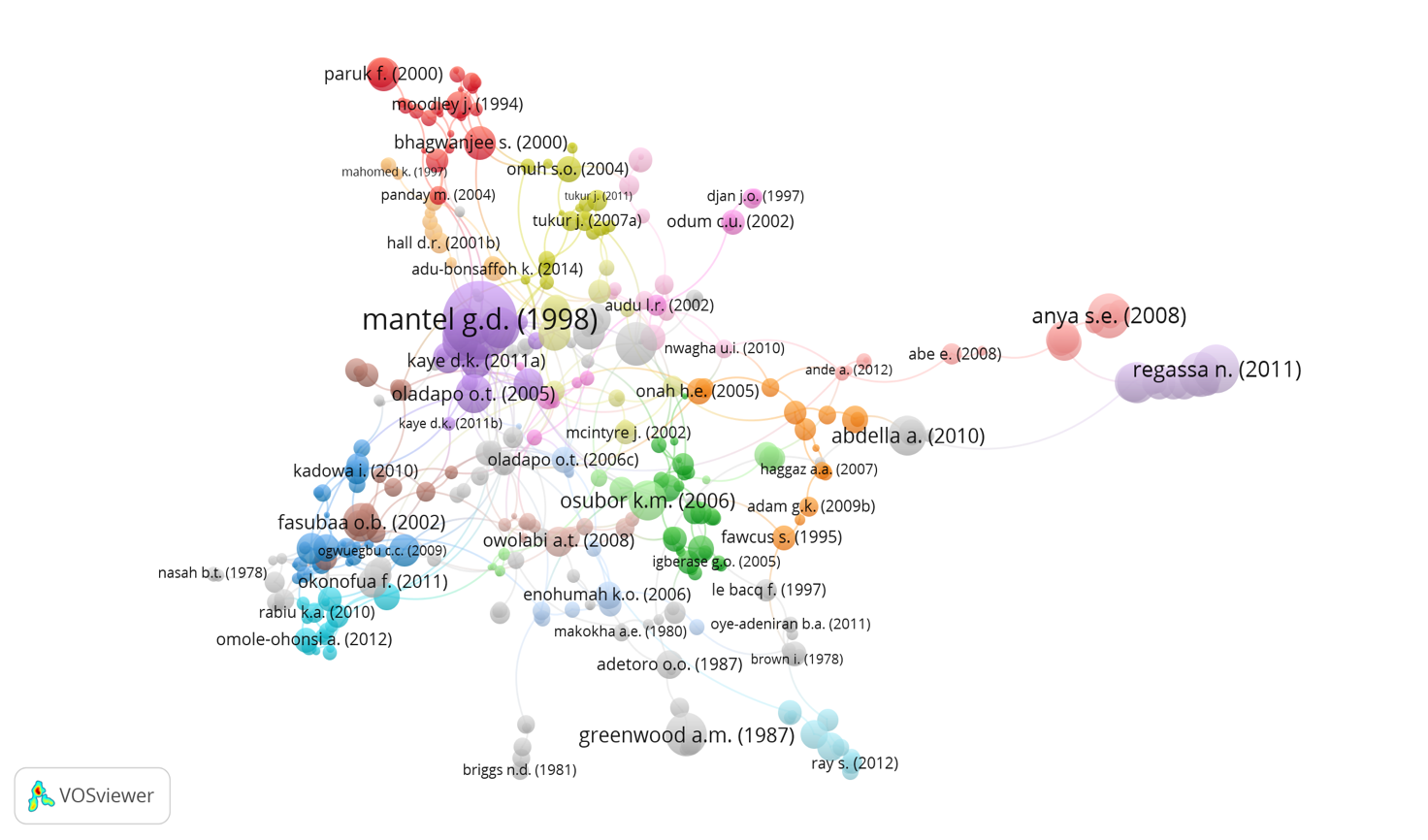


1. *Network visualization of influential publications from 1975-2014 (n = 974 Documents, retrieved from Scopus database).*


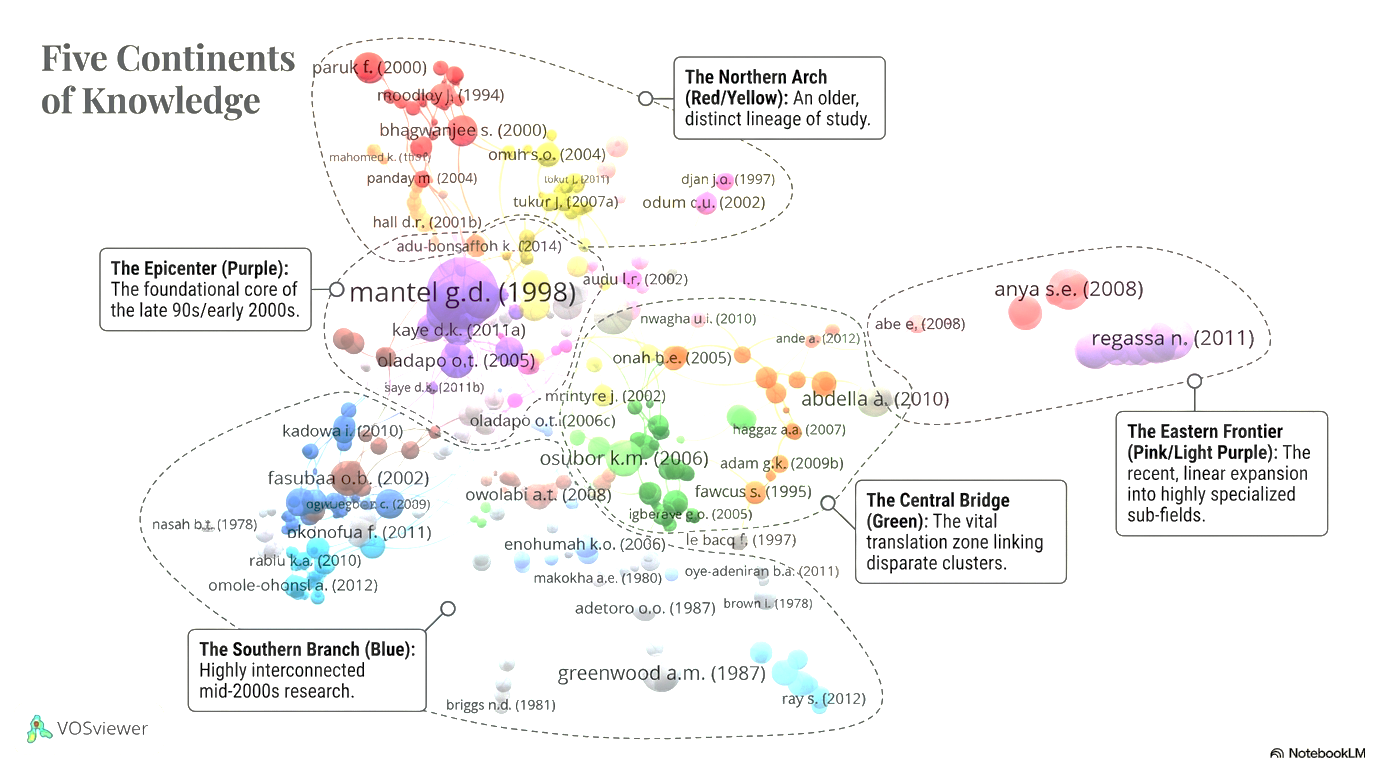


1. *A network visualization analysis of publications related to maternal mortality co-authored by African researcher from 1975-2014, n =974, from Scopus database).*


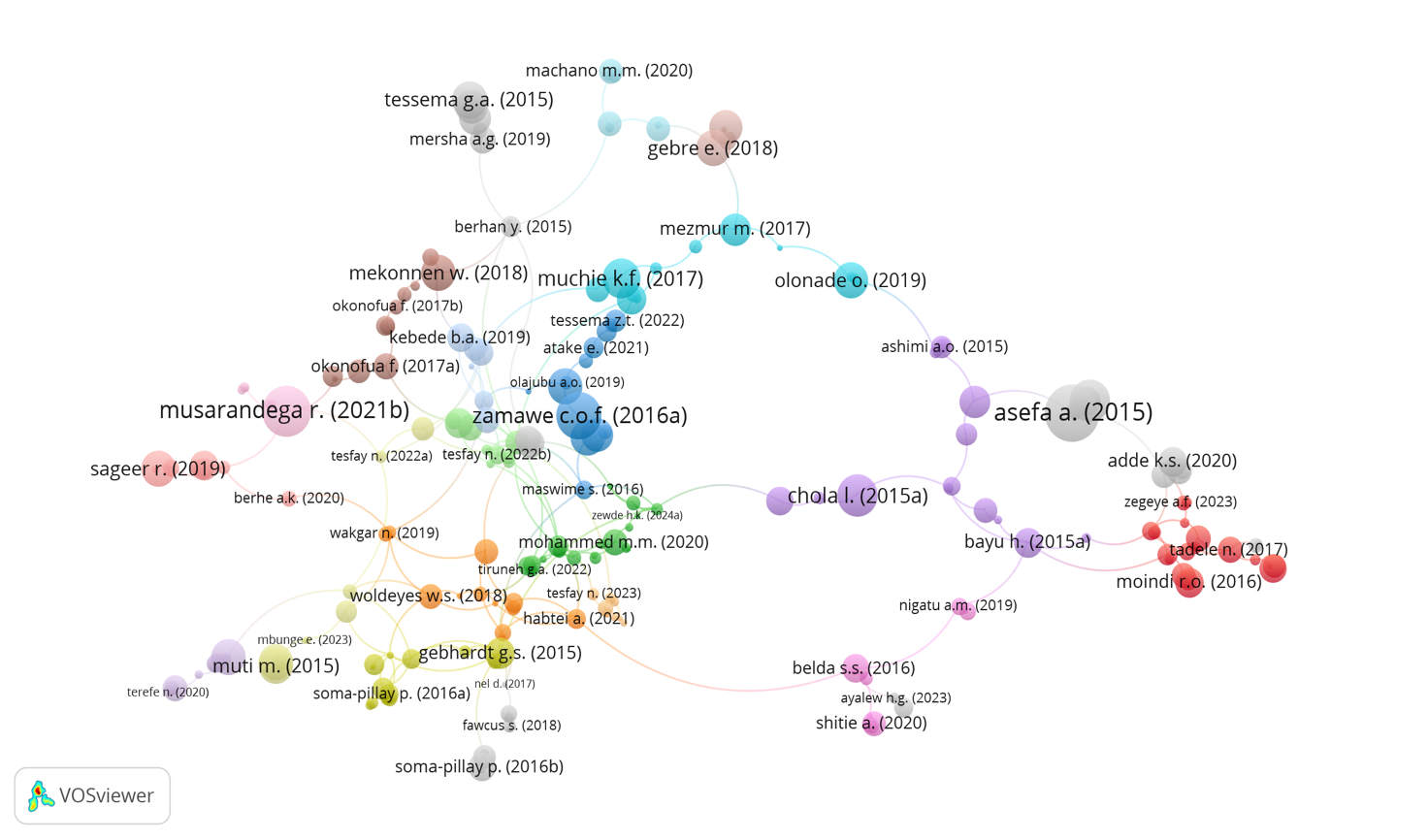


1. *Network visualization of influential publications from 2015-2024 (n = 1011 Documents, retrieved from Scopus database).*


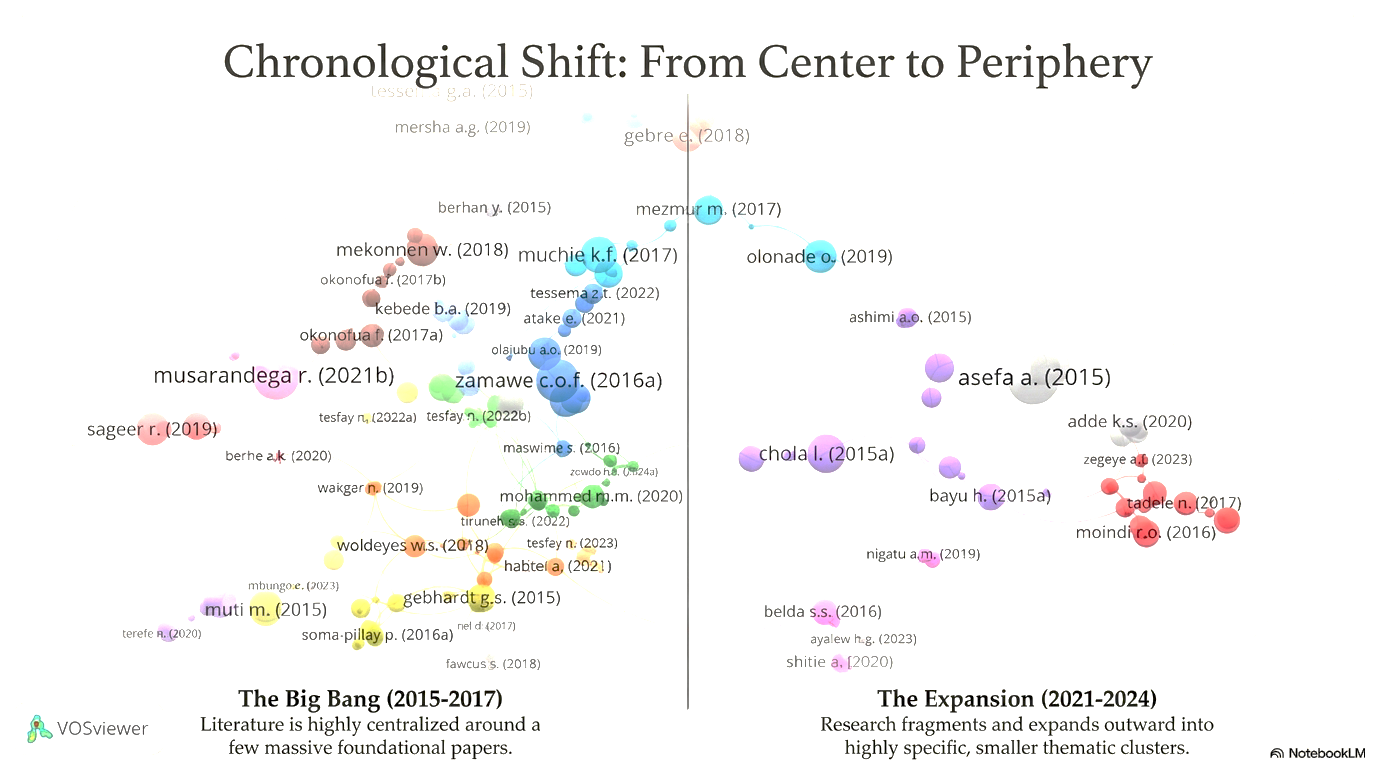


1. *A network visualization analysis of publications related to maternal mortality co-authored by African researcher from 2015-2024, n =1011, from Scopus database).*
